# Modifying and reacting to the environmental pH can drive bacterial interactions

**DOI:** 10.1371/journal.pbio.2004248

**Published:** 2018-03-14

**Authors:** Christoph Ratzke, Jeff Gore

**Affiliations:** Physics of Living Systems, Department of Physics, Massachusetts Institute of Technology, Cambridge, Massachusetts, United States of America; Synthetic and Systems Biology Unit, Hungary

## Abstract

Microbes usually exist in communities consisting of myriad different but interacting species. These interactions are typically mediated through environmental modifications; microbes change the environment by taking up resources and excreting metabolites, which affects the growth of both themselves and also other microbes. We show here that the way microbes modify their environment and react to it sets the interactions within single-species populations and also between different species. A very common environmental modification is a change of the environmental pH. We find experimentally that these pH changes create feedback loops that can determine the fate of bacterial populations; they can either facilitate or inhibit growth, and in extreme cases will cause extinction of the bacterial population. Understanding how single species change the pH and react to these changes allowed us to estimate their pairwise interaction outcomes. Those interactions lead to a set of generic interaction motifs—bistability, successive growth, extended suicide, and stabilization—that may be independent of which environmental parameter is modified and thus may reoccur in different microbial systems.

## Author summary

Microbes typically live alongside many other species in complex communities. These microbial communities are very important for us because they also live in and on our bodies and can determine our health and well-being. The composition and function of these communities, such as who is part of such a community and who is excluded, are decided by the interactions between the microbes. These microbial interactions can be driven by many different factors such as resource competition or toxin production. Although these factors are all different, the interactions are typically mediated through the environment; the microbes modify the environment, and they and other microbes have to live in this new environment. We show here that by understanding how microbes change and react to the environment, it is possible to understand and even predict their interactions. We believe that this way of thinking about microbial interactions will lead to a better understanding of more complex communities that are so important for our well-being.

## Introduction

Microbes thrive essentially everywhere on this planet, usually as part of complex multispecies communities [1]. The interactions between the microbes influence their growth and survival and thus the composition of those communities [2–8]. Although microbial interactions are of clear importance, only limited insights exist. They are often obtained from microbial systems in which a specific molecular mechanism causes a specific interaction, like mutualism caused by crossfeeding of amino acids, organic acids, and unknown substances [9–17]; crossprotection from antibiotics [18–21]; or competition by toxins [13,22–24].

Can we develop a more general understanding of microbial interactions? Despite the large number of possible types of interactions, they all have certain points in common because interactions are, in general, mediated through the environment. First, microbes change the environment by consuming resources and excreting metabolites. Second, these changes to the environment influence the growth and survival of both the microbe that originally altered the environment as well as other microbial species that are present. Therefore, the interaction between microbes may be set by how their metabolisms change the environment and react to those changes. A very important parameter for microbes is the pH, and different species prefer different pH values. Therefore, pH strongly influences the species composition in soil [25–29] or the human gut microbiome [30]. On the other hand, many biochemical reactions involve a turnover of protons, and therefore microbes also alter the pH around them. We chose the pH as a model environmental parameter—because it can be easily measured, manipulated, and buffered and is, moreover, important for all microbes—and show here that the way microbes modify the pH of their environment feeds back on them but also influences other microbes. This determines their growth behavior and the interactions between different bacterial species based upon how each species interacts with pH changes in the environment.

We show here, with the pH as a model parameter, that knowing the preferred environmental conditions of bacteria and how the bacteria manipulate the environment sets their interactions and allows us to predict the interactions between the microbes based on their metabolic properties. This approach may deliver a framework to understand and predict microbial interactions independent of the underlying molecular mechanism.

## Results

**Fig 1:**
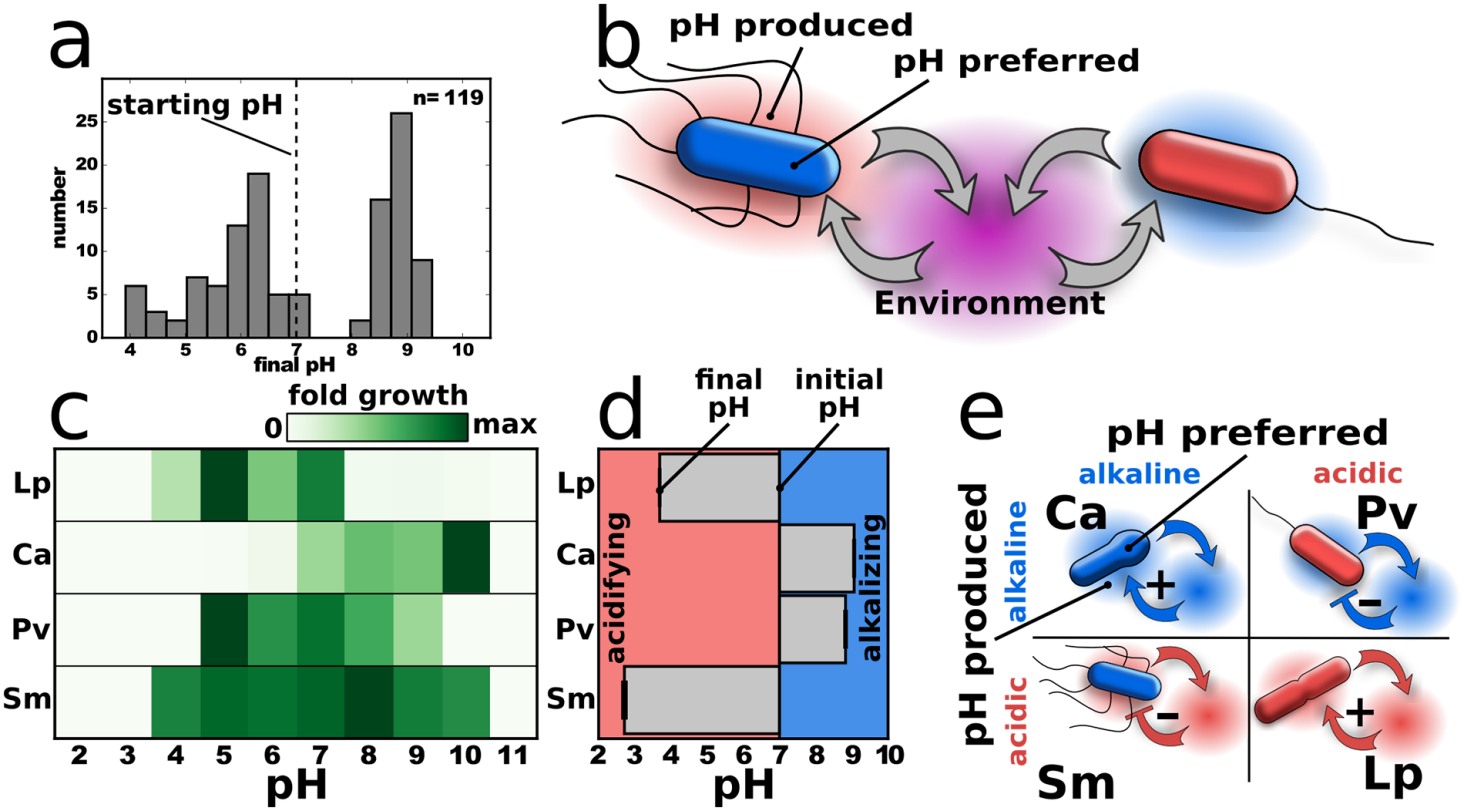
Bacteria modify the environment and react to it. (a) A collection of soil bacteria grown in a medium that contains urea and glucose can lower or increase the pH (initially set to pH 7, dashed line). The soil the microbes were isolated from has a buffer capacity similar to the experimental medium (S2 Fig). Also, growing the soil bacteria in Luria-Bertani medium causes pH changes (S2 Fig). (b) By changing the environment, bacteria influence themselves but also other microbes in the community. (c) *Lactobacillus plantarum* and *Pseudomonas veronii* prefer acidic, *Corynebacterium ammoniagenes* prefers alkaline, and *Serratia marcescens* has a slight preference towards alkaline environments. Fold growth in 24 h is shown. The bacteria were grown on buffered medium with low nutrients to minimize pH change during growth (Materials and methods and S2 Fig). (d) Starting at pH 7, *L*. *plantarum* and *S*. *marcescens* decrease and *C*. *ammoniagenes* and *P*. *veronii* increase the pH. Only little buffering, 10 g/L glucose and 8 g/L urea as substrates were used in (d). (e) Microbes can increase or decrease the pH (blue environment is alkaline, and red environment is acidic) and thus produce a more or less suitable environment for themselves. Blue bacteria prefer and/or tolerate alkaline and red acidic conditions. The soil bacteria in (a) were isolated from local soil, whereas the 4 species in (c) to (e) were obtained from a strain library (see Materials and methods for details). The data for this figure can be found in S1 Data. Ca, *Corynebacterium ammoniagenes*; Lp, *Lactobacillus plantarum*; Pv, *Pseudomonas veronii*; Sm, *Serratia marcescens*.

Growing a collection of soil microbes in a medium that contains 1% glucose as the main carbon source and 0.8% urea as the main nitrogen source leads to a change of the initial pH for nearly all tested bacteria (*n* = 119; for phylogenetic identity, see S1 Fig) (Fig 1a). One percent glucose lies within the range of carbohydrates in soil (0.1% [31] to 10% [32]), although the carbohydrates in soil are often more complex. Also, the bacterial densities that are reached in the experiments lie within the range that can be found in soil [33,34], and soil has even a slightly lower buffering capacity than our medium (S2 Fig). Microbial growth modulates the pH, but the pH is also known to strongly affect microbial growth [25,27,35,36]. In this way, microbes feed back on their own growth but also influence the growth conditions of other species that might be present (Fig 1b).

Microbes can lower or increase the pH, which may be beneficial or deleterious for their own growth. This leads to 4 possible combinations (Fig 1e), for which we identified an example species of each (Fig 1b, 1c and 1d; Materials and methods and S2 and S3 Figs). *Lactobacillus plantarum* is an anaerobic bacterium that produces lactic acid as metabolic product and thus lowers the pH but also prefers low pH values [37]. *Corynebacterium ammoniagenes* produces the enzyme urease that cleaves urea into ammonia and thus increases the pH [38]; at the same time, it prefers higher pH values. *Pseudomonas veronii* also increases the pH of the medium but prefers low pH values for growth. Finally, *Serratia marcescens* strongly lowers the pH [39] but better tolerates comparably higher pH values, with a slight optimum at around pH 8. As expected, the strength of the pH change depends on the amount of glucose and urea (S2 Fig) and can be tempered by adding buffer (S2 and S4 Figs). A pH change can also be first good and then bad for a microbe by first shifting the pH towards the optimum but then keeping on shifting it beyond it (S2 Fig); however, we focus here on the simple cases. Also, oxygen levels are changed by the bacteria but seem not to have a major influence on the system (S5 Fig). In summary, we find that microbial growth often leads to dramatic changes in the pH of the environment, and this pH change can promote or inhibit bacterial growth.

**Fig 2:**
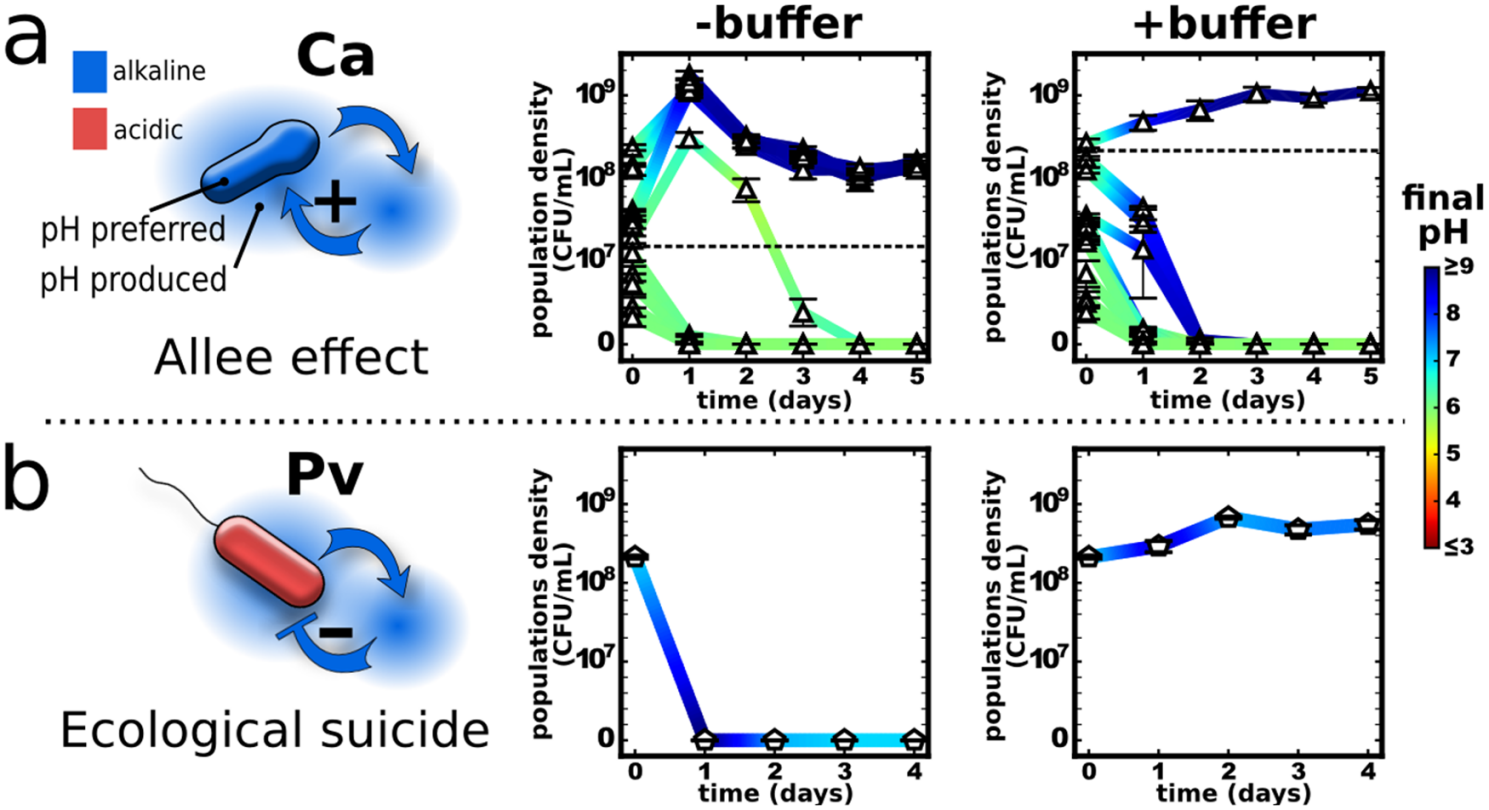
Single species can enhance or inhibit their own growth via changing the pH. The curves show bacterial density over time, and the color shows the pH. (a) *C*. *ammoniagenes* increases the pH and also prefers these higher pH values, leading to a minimal viable cell density required for survival. Increasing the buffer concentration from 10 mM (−buffer) to 100 mM (+buffer) phosphate makes it more difficult for *C*. *ammoniagenes* to alkalize the environment and therefore increases the minimal viable cell density. (b) *P*. *veronii* also increases the pH yet prefers low pH values. Indeed, *P*. *veronii* populations can change the environment so drastically that it causes the population to go extinct. Adding buffer tempers the pH change and thus allows for the survival of *P*. *veronii*. An Allee effect can also be found in *L*. *plantarum* and ecological suicide in *S*. *marcescens* (S8 Fig). Note that buffering often just slightly affects the final pH values (S2 Fig) but saves the population by delaying the pH change (as shown in S4 Fig and discussed in more detail in [35]). Linlog scale is used for the y-axis. The data for this figure can be found in S1 Data. Ca, *Corynebacterium ammoniagenes*; CFU, colony-forming unit; Pv, *Pseudomonas veronii*.

When the pH modification is beneficial for the bacteria, there is a positive feedback on their growth. The more bacteria there are, the stronger they can change the environment and thus the better they do. At adverse pH conditions, a sufficiently high cell density may therefore be needed to survive at all—an effect known as strong Allee effect [40–42]. Indeed, we observe such an effect: *C*. *ammoniagenes* promotes its own growth by alkalizing the environment, leading to a minimal starting cell density required for survival under daily batch culture with dilution (Fig 2a). Adding buffer (Fig 2a, right, S2 and S4 Figs) or lowering the nutrient concentration (S2 and S6 Figs) tempers the pH change and thus necessitates an even higher bacterial density for survival. Changes to the pH could therefore be a common mechanism of cooperative growth that leads to an Allee effect and an associated minimal viable population size.

Bacteria can also change the environment in a way that harms themselves, for example, by shifting the pH away from the growth optimum. In the most extreme case, the bacteria may make the environment so detrimental that it becomes deadly for them, an effect we call ecological suicide. Indeed, we find that *P*. *veronii*, which prefers lower pH, alkalizes the medium and thus causes its own extinction (Fig 2b). Again, adding buffer (Fig 2b, right) or lowering nutrient concentrations (S7 Fig) tempers the pH change and saves the population. It is therefore not the initial condition that kills the bacteria but the way the population changes the pH, which is further underlined by optical density (OD) measurements and following the colony-forming unit (CFU) over time (S7 Fig). The effect of ecological suicide is investigated in more detail in a separate manuscript [35].

Microbes that modify the environment not only affect their own growth but also other microbial species that may be present. In this way, the environmental modifications could drive interspecies interactions. We described previously (Fig 1) 4 different ways that a single species can change the environment and in turn be affected: lowering and increasing the pH and affecting the growth in positive or negative ways. Accordingly, there exist, in principle, 6 (4 choose 2) pairwise combinations of the 4 types in Fig 1d; however, 2 of them are symmetric cases of each other, leaving 4 unique interaction types (S10 Fig). We have seen that the microbial metabolism can determine the fate of a population by changing and reacting to the pH. Therefore, can we, in a similar way, understand and perhaps even anticipate possible outcomes of those 4 interactions based on the properties of the single species? To explore this question, we captured the essential elements of the pH interaction in mono- and coculture via a simple differential equation model (see S1 Text and S11 Fig).

$$\frac{\partial n_{a/b}}{\partial t}=n_{a/b}\left(1-\frac{n_{a/b}}{K}\right)\left(e^{\frac{-{\left(p-p_{pre\;fa/b}\right)}^{2}}{\sigma^{2}}}-\delta \right)$$ (1)

$$\frac{\partial p}{\partial t}=\left(c_{a}n_{a}+c_{b}n_{b}d\right)\left(\frac{p}{b}\left(2b-p\right)\right)$$ (2)

The bacteria densities n_a/b_ follow a logistic growth that saturates at the carrying capacity *K*. However, the overall growth rate also depends on the proton concentration, and growth becomes maximal at a preferred proton concentration, p_pref_. For the dependence of the growth on the proton concentration, we used a Gaussian function; however, the general properties of the system do not depend on the exact choice of this function (S1 Text). The further away the proton concentration is from the optimum of the species, the slower the bacteria grow and finally start to die, whereas δ sets the maximal death rate. The proton concentration p is changed by the bacteria, according to their density n_a/b_ and their strength in changing the proton concentration c_a/b_, which is set to +/− 0.1. Multiplication by a quadratic function ensures that the proton concentration stays within [0, 2b], which takes into account that the pH between inside and outside the bacteria can only be changed until the difference in chemical potential becomes too big [43]. The outcomes of the model should be regarded as qualitative. Stability analysis of this model supports the simulation outcomes shown in S1 Text. Adding a periodic dilution can affect the outcome at high dilution rates but does not qualitatively affect the results at sufficiently low dilution rates (S13 Fig). Moreover, a fuzzy logic–based model—that does not rely on defined functions—leads to very similar results; the outcomes are basically the same, but stabilization happens over a wider parameter range (S1 Text and S14 and S15 Figs).

**Fig 3:**
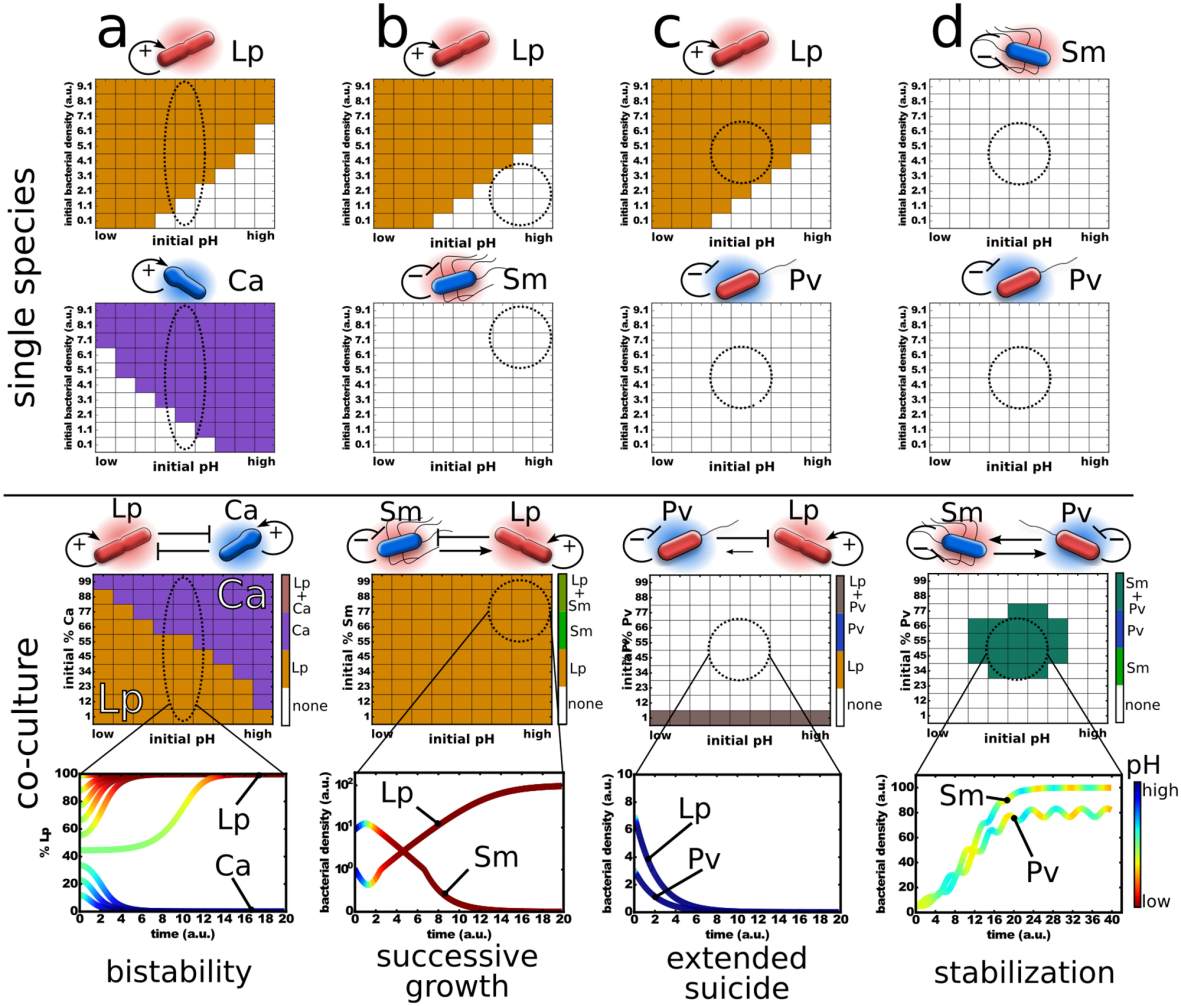
The metabolic properties of the different species may allow estimation of their interactions. A simple model based on differential equations was set up to qualitatively simulate the bacterial growth (see main text). The survival of the species at the end of the simulation for different initial parameter values are plotted as follows. A species that is extinct at the end of the simulation run either did not grow under these conditions, was outcompeted by another species, or went extinct by ecological suicide. The upper two rows of panels show the simulation outcome for the single species. The third row of panels shows the outcomes of the cocultures, for which representative time series of the phase diagrams marked by the dashed circles are shown in the bottom row. The “pH” scale reaches from low to high, which corresponds to a “proton concentration” of 10 to 0. σ was set to 4 and δ to 0.5. c_a/b_ was set to +/− 0.1, b to 5, and d as described in the S1 Text. (a) *L*. *plantarum* and *C*. *ammoniagenes* show bistability in coculture depending on the initial fraction and pH. (b) *L*. *plantarum* and *S*. *marcescens* show successive growth at high initial pH values, for which *L*. *plantarum* can only survive if the pH was first lowered by *S*. *marcescens*. Note that a high percentage of *S*. *marcescens* in the coculture panel (dashed circle) means low *S*. *marcescens* and high *L*. *plantarum* in the upper panels. (c) If *P*. *veronii* lowers the proton concentration by enough, it can kill itself and also *L*. *plantarum*, resulting in extended suicide. The coexistence at initial low ratios of *P*. *veronii* is caused by oscillatory dynamics as shown in S12 Fig. (d) *S*. *marcescens* and *P*. *veronii* can protect each other from ecological suicide and coexist, whereas they cannot survive on their own. The effect of varying interaction strength and initial conditions are shown in S11 Fig. We use the words bistability, successive growth, extended suicide, and stabilization merely to characterize the interaction outcomes and not any “intentions” of the bacteria. Linlog scale is used for the y-axis. Ca, *Corynebacterium ammoniagenes*; Lp, *Lactobacillus plantarum*; Pv, *Pseudomonas veronii*; Sm, *Serratia marcescens*.

For the single-species cases of *L*. *plantarum* and *C*. *ammoniagenes*, this model naturally gives an Allee effect—a minimal initial bacterial density is needed for survival depending on the initial pH value (Fig 3 and S11 Fig). In addition, when the species are changing the environment in a way that is bad for them (e.g., *P*. *veronii* and *S*. *marcescens*), the model yields ecological suicide in which the population dies under all starting conditions (Fig 3 and S11 Fig). Therefore, this simple model can capture the measured outcomes for the single species.

**Fig 4:**
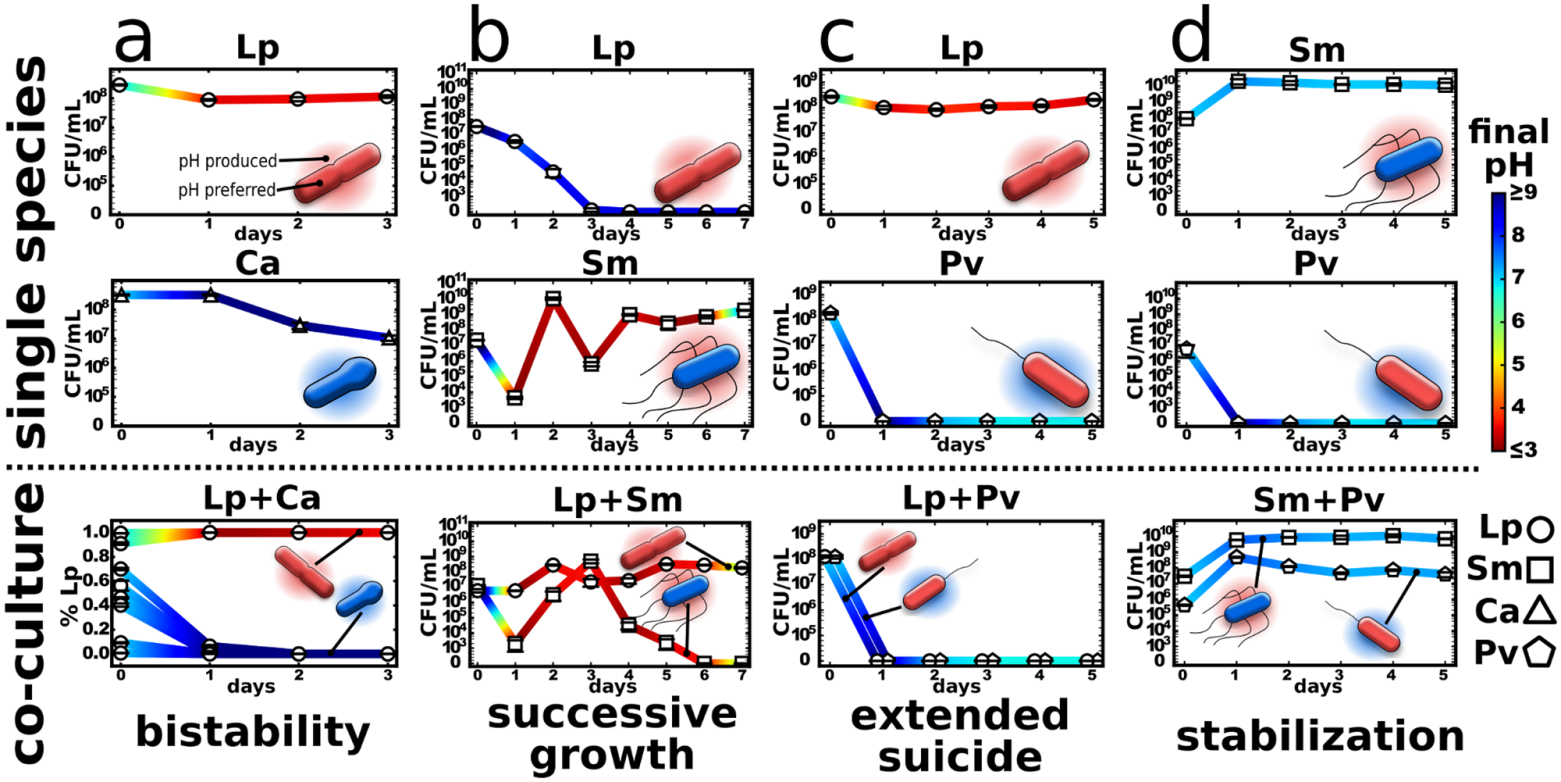
Modifying the environment drives interactions between microbes. Four different interaction types can be found depending on how the environmental changes act on the organisms themselves and each other. (a) *L*. *plantarum* and *C*. *ammoniagenes* produce bistability. (b) *S*. *marcescens* and *L*. *plantarum* show successive growth. (c) *P*. *veronii* commits extended suicide on *L*. *plantarum*. (d) *S*. *marcescens* can stabilize *P*. *veronii* when the medium is sufficiently buffered. For a more detailed description of the different interactions cases, see the main text. The media composition and protocols are slightly different for the different cases. See Materials and methods for details. We use the words bistability, successive growth, extended suicide, and stabilization merely to characterize the interactions outcomes and not any “intentions” of the bacteria. Linlog scale is used for the y-axis. The bacteria were diluted every 24 h into fresh media with a dilution factor of 1/100x (a and b) or 1/10x (c and d). The data for this figure can be found in S1 Data. Ca, *Corynebacterium ammoniagenes*; CFU, colony-forming unit; Lp, *Lactobacillus plantarum*; Pv, *Pseudomonas veronii*; Sm, *Serratia marcescens*.

We next explored whether the model can provide insight into the two species’ interactions. In particular, there is a question of whether just one environmental parameter—the pH—is sufficient to predict nontrivial outcomes of interspecies competition, neglecting all the other ways in which the species interact. In what follows, we focus on the outcomes predicted by the model that are marked by dashed circles in Fig 3. To test if those 4 outcomes can indeed be found in the experiments, the corresponding interaction pairs were grown with daily dilutions both as single species and in pairs under the conditions suggested from the model (Fig 4).

*L*. *plantarum* and *C*. *ammoniagenes* each modify the environment in a way that is good for themselves yet bad for the other species. Given this “antagonistic niche construction,” the model predicts that the coculture can yield bistability, in which only one species will survive, whereas the winner depends on the initial species abundances and initial pH (Fig 3a). Consistent with this prediction, we experimentally find bistability in the coculture of *L*. *plantarum* and *C*. *ammoniagenes*, with the initially abundant species driving the other species extinct (Fig 4a and S11 Fig). As expected, removing glucose and urea from the medium prevents pH changes and thus also removes the forces leading to bistability, which results in coexistence between the two species (S16 Fig). Two species that are attempting to modify the environment in incompatible ways can therefore lead to the emergent phenomenon of bistability.

*L*. *plantarum* does not tolerate high pH values, whereas *S*. *marcescens* can grow at high pH but subsequently acidifies the environment and kills itself (S8 and S11 Figs). This raises the question of whether *S*. *marcescens* can aid *L*. *plantarum* by lowering an initially high pH, thus allowing *L*. *plantarum* to survive. Indeed, our model suggests that it may be possible to observe successive growth, in which *S*. *marcescens* grows first and lowers the pH, thus allowing *L*. *plantarum* to survive despite the fact that the starting pH would have been lethal for *L*. *plantarum* (Fig 3b and S11 Fig). We can indeed find this interaction motif experimentally. At an initially high pH, *L*. *plantarum* can only survive if *S*. *marcescens* helps to modify the environment (Fig 4b). However, contrary to the simulation, *S*. *marcescens* alone does not experience ecological suicide in the experiment, likely because the high starting pH prevents *S*. *marcescens* from lowering the pH too rapidly. Indeed, at lower starting pH values, *S*. *marcescens* shows ecological suicide (S8 and S11 Figs). Adding buffer or removing nutrient causes only moderate acidification and thus kills *L*. *plantarum* but allows *S*. *marcescens* to survive (S17 Fig). In successive growth, *L*. *plantarum* can only establish growth after *S*. *marcescens* has prepared the ground for it, a well-understood phenomenon in plant ecology [44,45].

Above, we considered the case of a species transiently helping another species to survive, raising the question of whether it might also be possible to observe the opposite situation, in which pH modification leads to ecological suicide and kills the interaction partner with it. Indeed, our model predicts that this “extended suicide” could be present for the *L*. *plantarum*/*P*. *veronii* pair over a wide range of cell densities and starting pH values (Fig 3c). To explore this prediction of our model experimentally, we utilize *P*. *veronii*, which commits ecological suicide via alkalization (Fig 2b), together with *L*. *plantarum*, which dies in alkaline conditions (Fig 2a). Once again consistent with the predictions of our simple model, we successfully observed such an extended suicide, in which the extreme alkalization of the media by *P*. *veronii* led to the death of both *P*. *veronii* and *L*. *plantarum* (Fig 4c). The addition of buffer tempers this alkalization sufficiently to stop *P*. *veronii* from suicide but still allows it to outcompete *L*. *plantarum* (S18 Fig). However, if *L*. *plantarum* were more effective at acidifying the media, then coexistence might have been possible (S11 Fig). We therefore find that ecological suicide can also have a negative impact on other present species.

Finally, the model suggests that it may be possible for 2 species to help each other avoid ecological suicide (Fig 3d). This would correspond to an obligatory mutualism, in which the opposing pH changes cancel each other and therefore allow for the stable coexistence of the 2 species in an environment in which neither species could survive on its own. However, this stabilization is only observed in the model in a rather small parameter range, which suggests that finding it experimentally may be difficult. Indeed, experimentally, we could not find conditions in which *P*. *veronii* and *S*. *marcescens* form the most extreme form of stabilization, in which each species alone results in ecological suicide yet together they survive. However, we did observe a situation in which *P*. *veronii* alone causes ecological suicide yet *S*. *marcescens* can stabilize the pH and allow both species to survive and coexist (Fig 4d; see Materials and methods for details). Species that harm themselves through their environmental modifications can therefore benefit from other species in the environment.

## Discussion

Although bacterial interactions are commonly regarded as a very complex business, we found here a surprising simplicity. By studying how bacteria change their environment and react to it, we were able to understand a variety of interactions within populations as well as interactions between different species. Although the interaction outcomes were quite different, they were all mediated by a single environmental parameter—the pH—which shows that even a rather complex set of interactions can be mediated through the same environmental parameter. This significantly simplifies the situation and gives hope that even more complex microbial communities may be tractable. Moreover, the pH is a very general parameter that essentially all microbes influence and depend on. Indeed, the pH could be identified as a major driver of bacterial interactions in nature in several cases like the oral [46,47] or gut microbiome [48,49]. Many microbes are known to show an Allee effect by cooperatively secreting “public goods”—like enzymes that break down complex sugars [50–53]. However, in our system, the Allee effect was mediated by the pH without the necessity of a specialized enzymatic machinery. This suggests that cooperation may be easier to achieve and more widespread than is often assumed.

Cooperation is a very important collective phenomenon, in which organisms work together to achieve something they could not on their own. However, we could also find a different type of collective action with which bacteria collectively deteriorate their environment and thus cause their own extinction—the ecological suicide. We discuss this surprising effect in more depth in a separate manuscript [35].

After understanding the interactions within a population as mediated through the environment, we went a step further and applied the same reasoning to interspecies interactions. Surprisingly, a simple model could quite accurately forecast the interaction motifs that we later found experimentally: bistability, successive growth, extended suicide, and stabilization—all driven by pH modifications. In our model (Fig 3), the pH could be replaced by any other environmental parameter or a combination of parameters that is affected by and affects microbes—like oxygen or metabolite concentrations. Therefore, describing microbial interactions as a combination of modifying the environment and reacting to it may give a general framework for microbial interactions independent of the exact underlying biochemical mechanism. And indeed, several examples for the described interaction motifs have been found in other studies. Bistability has been observed in the gut microbiome [54], successive growth in the colonization of chitin particles [55] or the human gut [56], and stabilization in a variety of crossfeeding and crossprotection metabolisms [11,18]. To our knowledge, ecological suicide and thus also extended suicide are not yet described in microbes. However, a self-inflicted decline of populations could be found in several macro-organisms [57–59]. In many cases, microbial interactions may not be driven by a single parameter—the pH in our case—but a set of multiple parameters. However, also in these cases, the interactions are mediated by modifying and reacting to the environment. Correspondingly, our framework can be used and expanded towards multivariate systems and may thus lay the basis to understand more complex microbial interactions.

## Materials and methods

All chemicals were purchased from Sigma Aldrich (St. Louis, MO) if not stated otherwise.

### Bacteria

#### Strains isolated from soil

The 119 bacterial strains (used in Fig 1a and S2 Fig) were isolated from a single grain of soil collected in September 2015 in Cambridge, Massachusetts, USA. The grain weighed approximately 1 mg and was handled using sterile technique. The grain was washed in phosphate-buffered saline (PBS), and serial dilutions of the supernatant were plated on nutrient agar (0.3% yeast extract, 0.5% peptone, 1.5% bacto agar) and incubated for 48 h at room temperature. Colonies with maximal variety in colony morphology were chosen. Isolated colonies were sampled and cultured at room temperature in 5 mL nutrient broth (0.3% yeast extract, 0.5% peptone) for 48 h. To ensure purity, the liquid cultures of the isolates were diluted in PBS and plated on nutrient agar. Single colonies picked from these plates were once again grown in nutrient broth for 48 h at room temperature, and the resulting stocks were stored in 20% glycerol at −80 °C. The 16S rRNA gene was sequenced via Sanger sequencing of DNA extracted from glycerol stocks carried out at GENEWIZ (South Plainfield, NJ). Sequencing was performed in both directions using the company’s proprietary universal 16S rRNA primers, yielding assembled sequences approximately 1,100 nt in usable length. Some of those strains have been investigated in more detail in [60]. A phylogenetic tree of the bacteria is shown in S1 Fig.

#### Lab strains

The 4 strains that were used in all experiments except the ones shown in Fig 1a and S2 Fig were purchased from ATCC (Manassas, VA): *L*. *plantarum* (ATCC 8014), *C*. *ammoniagenes* (ATCC 6871), *P*. *veronii* (ATCC 700474), and *S*. *marcescens* (ATCC#13880).

### Buffer

For precultures of the bacteria, the basic buffer recipe was 10 g/L yeast extract (Becton Dickinson, Franklin Lakes, NJ) and 10 g/L soytone (Becton Dickinson). We refer to that buffer as 1xNutrient medium. For the washing steps and the experiment itself, the medium contained 1 g/L yeast extract and 1 g/L soytone, 0.1 mM CaCl_2_, 2 mM MgCl_2_, 4 mg/L NiSO_4_, 50 mg/L MnCl_2_, and 1x Trace Metals Mixture (Teknova, Hollister, CA). We refer to that buffer as base buffer. For the different bacteria, the initial pH was either 6 or 7, and it was supplemented with phosphate buffer and/or glucose and urea as outlined in the specific experiments below. The glucose and urea were added freshly every day directly before starting the experiments, to avoid degradation of the urea. The usual concentrations were 10 g/L glucose and 8 g/L urea; deviations from that are described for the single experiments below.

All media were filter sterilized.

### Estimation of CFUs

To estimate the population density of living bacteria in the different experiments, we used colony counting. At the end of every growth cycle, a dilution row of the bacteria was made by diluting them 7 times 1/10x in PBS (Corning, NY). With a 96-well pipettor (Viaflo 96, Integra Biosciences, Hudson, NH), 10 μL of every well for every dilution step was transferred to an agar plate (Tryptic Soy Broth [Teknova], 2.5% Agar [Becton Dickinson], and 50 mg/L MnCl_2_—in the case of *L*. *plantarum*—were plated) with 150-mm diameter. The droplets were allowed to dry in, and the plates were incubated at 30 °C for 1 to 2 days until clear colonies were visible. The different dilution steps made sure that a dilution could be found that allows counting of single separated colonies. The different bacteria could be distinguished by their colony morphology (S3 Fig).

### pH measurements

To measure the pH directly in the bacterial growth culture at the end of each growth cycle, a pH microelectrode (N6000BNC; SI Analytics, Weilheim, Germany) was used. The bacterial cultures were transferred into 96-well PCR plates (VWR, Radnor, PA) that allowed us to measure pH values in less than 200 μL.

### Bacterial culture

All cultures were incubated at 30 °C. The precultures were done in 5 mL medium in 50 mL culture tubes (Falcon/Becton Dickinson,) overnight in the 1xNutrient described above, with different pH values as outlined below. The shaking speed was 250 rpm on a New Brunswick Innova 2100 shaker (Eppendorf, Hauppauge, NY); the lids of the Falcon tubes were only slightly screwed on to allow gas exchange. The experiments were all done in 96-deepwell plates (Eppendorf) covered with two sterile AearaSeal adhesive sealing films (Excell Scientific, Victorville, CA); the plates were shaken at 1,350 rpm on Heidolph platform shakers (Titramax 100, Heidolph North America, Elk Grove Village, IL). To avoid evaporation, the shakers were covered with a custom-made polyacryl box (Wetinator 2000) with small water reservoirs placed within.

### Single-species experiments

#### pH modification by isolated soil strains

The 119 bacterial strains were pregrown in TSB medium (Teknova) for 24 h at RT in deepwell plates. The bacteria were diluted 1/100 into base medium with 10 mM phosphate, 8 g/L urea, and 10 g/L glucose and grown for another 24 h. The pH was measured in the wells that showed a final OD of >0.2 (100 μL in 96-well flat-bottom plates). A similar experiment was done by growing the bacteria in LB medium (Becton Dickinson).

#### pH modification by the four lab strains

*L*. *plantarum* and *C*. *ammoniagenes* were precultured in 1xNutrient—pH 6 (*L*. *plantarum*) or 7 (*C*. *ammoniagenes*)—and *S*. *marcescens* and *P*. *veronii* were precultured in 1xNutrient—pH 7, 100 mM phosphate—overnight at 30 °C. The next day, *P*. *veronii* was diluted 1/100x and *S*. *marcescens* 1/4,000x into the same medium. Upon *P*. *veronii* and *S*. *marcescens* reaching OD/cm 2, all four precultures were spun down with 4,000 rpm for 2 min on an Eppendorf Tabletop centrifuge (Centrifuge 5810, with rotor A-4-81) and washed two times with base buffer with 10 mM phosphate, pH 7. The OD/cm was adjusted to 2. The bacteria were diluted 1/100x into 200 μL base—pH 7, 10 mM phosphate—with 10 g/L glucose and 8 g/L urea in 96-deepwell plates (Eppendorf); they were then grown at 30 °C and 1,350 rpm shaking speed for 24 h. Afterwards, the pH was measured in every well with the micro pH electrode (N6000BNC; SI Analytics).

### Growth of bacteria at different pH values

Preculture and cell preparation were done as described for “pH modification by the four lab strains.” After washing the cells, the bacteria were diluted 1/100x into 96-deepwell plates with 200 μL of base—pH 7, 100 mM phosphate—in each well with a pH varying in integers from 2 to 11. The initial CFUs were estimated by plating the bacteria that should be incubated at pH 7 at the beginning of the incubation. After 24 h at 30 °C and 1,350 rpm shaking speed, the bacteria were plated again, and the final pH was measured (S2 Fig). The final CFUs were divided by the initial CFUs, and the maximum was normalized to 1 to make the values comparable (Fig 1B).

### Allee effect

The experiments were performed with *C*. *ammoniagenes*, which is urease active and thus capable of cleaving urea into ammonia [38]. The bacteria were precultured as described in “pH modification by the four lab strains” above except that the bacteria were washed with base medium with pH 6. The bacterial solution was split into two equal amounts, spun down, and resuspended with 2.5 mL of base pH 6 with either 10 mM or 100 mM phosphate. The OD/cm was adjusted to 2. For the experiments, 96-deepwell plates (Eppendorf) were used.

The experiment was performed in 3 different media: (i) base, pH 6 with 10 mM phosphate (low-nutrient condition); (ii) base pH 6 with 10 mM phosphate, 10 g/L glucose, and 8 g/L urea (low-buffer conditions); and (iii) base pH 6 with 100 mM phosphate, 10 g/L glucose, and 8 g/L urea (high-buffer conditions). The final volume was 100 μL per well. In the first row, 270 μL of media were prepared and 30 μL of OD/cm = 2 bacteria solutions were added. Starting from this row, 2/3x (200 μL into 100 μL) dilutions were performed into the next rows. This way, a gradient of initial cell densities was generated. From the last row, 200 μL of bacteria solution were removed in the end to obtain 100 μL in each well. The bacteria were cultivated at 30 °C shaking at 1,350 rpm (Titramax 100, Heidolph North America). A 1/10x dilution into fresh medium was performed every 24 h.

The CFU was estimated as described above immediately after setting up the experiments and after every 24-h growth cycle. Moreover, the pH of the medium was measured at the end of the growth cycles. For every condition, we used 4 replicates.

### Ecological suicide

For this experiment, *P*. *veronii* was used, a bacterium that can alkalize the environment by urea cleavage but itself prefers lower pH for growth. The preculture and cell preparation were done as described in “pH modification by the four lab strains.” The 96-deepwell plates were prepared by adding 200 μL base with 10 mM phosphate—with or without 10 g/L glucose and 8 g/L urea (high- and low-nutrient conditions)—or base with 100 mM phosphate—with 10 g/L glucose and 8 g/L urea (high-buffer condition)—to the 96-deepwell plates. The bacteria were added by 1/100x dilution. The plate was incubated at 30 °C, 1,350 rpm shaking. Every 24 h, the bacteria were diluted 1/100x into fresh medium, the CFU estimated, and the pH measured. For every condition, we measured 4 replicates.

### Interspecies interactions

#### Bistability

*C*. *ammoniagenes* and *L*. *plantarum* were used here. Both species change the pH in a direction that they themselves tolerate but the other species does not tolerate. *C*. *ammoniagenes* and *L*. *plantarum* were precultured for 17 h in 1xNutrient, pH 7 (*C*. *ammoniagenes*) or pH 6 (*L*. *plantarum*). The overnight cultures were washed with base with 10 mM phosphate, pH 7, two times and the OD/cm adjusted to 2. The two bacterial species were mixed with ratios 1%, 10%, 30%, 45%, 55%, 70%, 90%, and 99%, keeping the sum OD/cm at 2. The bacterial mixture was diluted 1/100x into 96-deepwell plates containing 200 μL base, with 10 mM phosphate with/without 10 g/L glucose and 8 g/L urea. The starting pH was 7 for the medium without glucose and urea and 6, 6.5, and 7 for the medium with glucose and urea. The cultures were incubated at 30 °C and shaken at 1,350 rpm. Every 24 h, the CFU and pH were measured and the cultures were diluted 1/100x into fresh medium. For the CFU counting, the dilutions of the cultures were plated onto TSB agar plates as described above. However, every dilution was plated twice—once on a TSB agar plate with pH 5 and once with pH 10. At pH 5, only *L*. *plantarum* could grow and at pH 10, only *C*. *ammoniagenes*; this way the bacteria could be easily distinguished. This was especially helpful because the colony morphology of *C*. *ammoniagenes* and *L*. *plantarum* is rather similar (S3 Fig). For every condition, we measured 3 replicates for the mixed culture and 8 replicates for the single species.

### Extended suicide

*L*. *plantarum* and *P*. *veronii* were used for this experiment. *L*. *plantarum* decreases the pH and likes low pH, whereas *P*. *veronii* increases the pH but prefers lower pH values. The preculture and cell preparation were done as described in “pH modification by the four lab strains” above. The experiment was done in 96-deepwell plates, each well containing 200 μL base, pH 7, with 10 mM or 100 mM phosphate and 10 g/L glucose and 8 g/L urea. The bacterial solution was diluted 1/100x (start OD/cm = 0.02) into the 96-deepwell plates. The plates were incubated at 30 °C with 1,350 rpm shaking speed. Every 24 h, the CFU was estimated by plating, the pH was measured, and the bacterial culture was diluted 1/100x into fresh medium. We measured 8 replicates for every condition.

### Successive growth

*L*. *plantarum* and *S*. *marcescens* were used for this experiment. The preculture and cell preparation were done as described in “pH modification by the four lab strains” above. *S*. *marcescens* and *L*. *plantarum* were mixed 1/1, resulting in a sum OD/cm of 2. The experiment was done in base with pH 10.2 and 10 mM phosphate with and without 10 g/L glucose, or 100 mM phosphate with 10 g/L glucose. The glucose was added and the pH adjusted directly before setting up the experiments, followed by filtering the media. The bacteria were diluted 1/100x into the measurement medium (= 0.02 OD/cm) and diluted 1/10x after every 24-h cycle together with the measurement of the CFU and pH. We measured 8 replicates for every condition.

### Stabilization

The preculture and cell preparation of *S*. *marcescens* and *P*. *veronii* were done as described in “pH modification by the four lab strains” above. The bacteria were mixed 1/1 (vol/vol). The experiment was performed in 96-deepwell plates with each well containing 200 μL of base with 65 mM phosphate, 10 g/L glucose, and 8 g/L urea, pH 7. The bacterial mixture was diluted 1/100x into each well, and the culture was incubated at 30 °C and shaken at 1,350 rpm. Every 24 h, the bacteria were diluted 1/10x into fresh medium, and the bacteria were diluted 7 times 1/10x in PBS buffer. For CFU measurement,150 μL of the 10^−2^, 10^−4^, and 10^−6^ dilutions were plated on TSB—2.5% agar 100 mm plates, each well on a full plate. Also, the pH for every well was measured. We measured 3 replicates for every condition.

## Supporting information

S1 Fig Species identities of the soil species of Fig 1a. A total of 119 strains were used for Fig 1a, and the 16S sequences of those strains were sequenced, which failed for a series of strains. The remaining 16S sequences were aligned with MuscleWS [61], and a Tree was constructed via Neighbour Joining [62]. The similarity matrix was calculated from the PID between the sequences after multiple sequence alignment. Nan means that the species could not be assigned likely because the underlying 16S rRNA sequence was too short. The alignment for the tree is provided as S3_Data. PID, percentage identity.
(EPS)

S2 Fig The final pH after microbial growth depends on available nutrient and buffering. (A) The higher the nutrient concentration, the stronger the pH change. (B) Buffer hinders the pH change. In the case of *S*. *marcescens*, there is no pH shift at all at high-buffer concentrations. Base buffer with 10 g/L glucose and 8 g/L urea. Bacteria were grown in the media for 24 h. Error bars show SEM but can barely be seen. In (A), 10 mM phosphate buffer was used. (C) The four species were grown in base, 100 mM phosphate with different initial pH values to test how their growth rate depends on the pH (Fig 1B). The absence of glucose and urea and the presence of buffer lead to rather small pH changes in growth. However, the high starting pH values drop significantly over time, which is likely caused by dissolution of CO_2_ in the growth medium (see S7 Fig). (D) Simulation of microbial growth illustrates how pH changes can lead to death of the population. The simulation was done as described in the main text and in the Supporting information below. The p_pref_ is set to 3.5. The initial proton concentration is set to 1. The bacteria increase the proton concentrations. In this way, they first facilitate their own growth but then keep on changing the proton concentration and kill themselves. (E) The soil bacteria from main text Fig 1A also show a pH change in Luria-Bertani medium. Again, the pH was measured after 24 h of growth of the soil bacteria in the medium. (F) The buffering capacity of the soil that the strains in Fig 1A were isolated from is lower than that of base medium, which means that pH effects in soil may be even more pronounced than in that medium. The data for this figure can be found in S2 Data. p_pref_, preferred proton concentration.
(PNG)

S3 Fig Colonies of the four different bacterial species. The colony color and morphology allow us to distinguish them pretty well, with the exception of *L*. *plantarum* and *C*. *ammoniagenes*. Although *L*. *plantarum* can be recognized because it is more translucent than *C*. *ammoniagenes*, *C*. *ammoniagenes* and *L*. *plantarum* were distinguished by plating them on agar plates with pH 4 and 10, respectively. *L*. *plantarum* only grows on the pH 4 plates and *C*. *ammoniagenes* only on the pH 10 plates.
(PNG)

S4 Fig Adding buffer delays the time of the pH change. *C*. *ammoniagenes* and *P*. *veronii* were grown in base medium, 10 g/L glucose, 8 g/L urea, and with 10 or 100 mM phosphate, respectively. The change of the pH was followed in real time by measuring the ratio of the pH-dependent fluorophore fluorescein and the pH-independent fluorophore TFPP. Both dyes were enclosed in nanobeads as described in [5]. The earlier the pH change happens, the longer the bacteria are exposed to the—for them—either benign (*C*. *ammoniagenes*) or detrimental (*P*. *veronii*) environment, which either increases or decreases their fitness as shown in Fig 2. The lines show the mean of 8 replicas, and the shaded region shows SEM. The data for this figure can be found in S2 Data. TFPP, tetrakis(pentafluorophenyl) porphyrin.
(PNG)

S5 Fig Oxygen levels in bacterial cultures. (A) Bacteria were grown in base medium with 100 mM phosphate buffer, pH 7 supplemented with 10 g/L glucose and 8g/L urea, at 30 °C in 200 μL volumes in deepwell plates with 1,350 rpm shaking and thus just like the samples for the other experiments. The oxygen content was measured with the extracellular oxygen consumption assay (ab197243; Abcam, Cambridge, MA). The higher the fluorescence signal, the less oxygen is in the sample. Oxygenated medium is just the medium without bacteria but otherwise treated like the cultures; deoxygenated medium is medium with 2 M sodium sulfite, which reacts with oxygen. As can be seen, oxygen depletion by the bacteria is moderate by most bacteria, with the exception of *S*. *marcescens*, which produces a rather strong anaerobic environment. This depletion of oxygen goes in line with a rather high cell density. *S*. *marcescens* was used in the experiments for successive growth with *L*. *plantarum* and stabilization with *P*. *veronii*. *S*. *marcescens* may support *L*. *plantarum* not only by lowering the pH but also depleting O_2_. Moreover, this oxygen depletion may also destabilize the proposed coexistence between *S*. *marcescens* and *P*. *veronii* and thus is a possible reason that we had difficulties finding it experimentally. The strong error for the OD of *S*. *marcescens* is likely caused by clumping of the cells, which disturbs the OD measurement. (B) The bacteria were grown for 24 h in base medium with 100 mM phosphate buffer and 10 g/L glucose and 8g/L urea at RT in 200 μL in deep-well plates either with normal aeration or in an anaerobic chamber containing 5% hydrogen, 20% carbon dioxide, and 75% nitrogen as atmosphere. The OD600 was measured at the beginning and end of the experiment to obtain the fold growth of the bacteria. The influence of oxygen on the growth is surprisingly small, with only *C*. *ammoniagenes* being strongly inhibited by the absence of oxygen. *L*. *plantarum* is an anaerobic bacterium and thus profits from the absence of oxygen. The data for this figure can be found in S2 Data. OD, optical density; RT, room temperature.
(PNG)

S6 Fig Low-nutrient conditions do not allow for pH change and thus cause *C*. *ammoniagenes* to go extinct. (A) *C*. *ammoniagenes* in base buffer without additional urea or glucose and a 1/10x dilution every 24 h. Starting at pH 6, the bacteria cannot sufficiently change the pH and thus go extinct. (B) At the same conditions but a starting pH of 7, all populations survive independent of the initial cell density (for B, the pH change was not recorded). The data for this figure can be found in S2 Data.
(PNG)

S7 Fig Ecological suicide of *P*. *veronii*. (A) In the absence of glucose and urea, *P*. *veronii* is not able to increase the pH drastically and thus does not kill itself. However, there is a small increase in pH, which is likely caused by the fact that the base medium contains peptides as carbon sources. In this case, excess ammonia is excreted and increases the pH (see S1 Fig). Daily dilution of 1/100x. (B) Under daily batch culture with dilution (1/100x), the OD at high-nutrient concentrations first increases and then drops to zero. In contrast, the CFU drops to zero after the first day of growth as a result of ecological suicide (Fig 2b). The OD is measuring light scattering and thus the presence of cells regardless of whether they are dead or alive. However, CFU just measures the living cells that are able to form colonies when plated on agar. Therefore, after the first day, we have zero living but a high number of dead cells. This shows that the bacteria could initially grow but later died out. Increased buffering of the media allows the population to survive multiple growth-dilution cycles, and the OD remains high (Fig 2b). (C) Ecological suicide of *P*. *veronii* can be followed with high temporal resolution, such that initially the CFU increases, but as the pH increases, the CFU starts to drop until the population goes extinct. Initially, the CFU increases, but as the pH increases, they start to drop till they go extinct completely. This phenomenon will be discussed in more detail in a separate publication. The data for this figure can be found in S2 Data. CFU, colony-forming unit; OD, optical density.
(PNG)

S8 Fig Allee effect of *L*. *plantarum* and ecological suicide of *S*. *marcescens*. (A) Starting at a high pH of 10.2, *L*. *plantarum* also shows a density-dependent fitness in media containing urea and glucose. Estimating the fold growth after 24 h only at initial high cell densities, a net growth could be achieved; at low cell densities, the bacteria die. Adding more buffer and thus making pH change more difficult leads to death at all initial cell densities. (B) *S*. *marcescens* strongly acidifies the environment and thus causes cell death (again the fold growth within 24 h was measured), but in this case, the addition of buffer leads to positive net growth—which shows that suicide is indeed caused by the pH change. The data for this figure can be found in S2 Data.
(PNG)

S9 Fig Media that start with high pH undergo a spontaneous decrease in pH that is independent of microbial activity. Base buffer with 10 mM phosphate was adjusted to a series of different initial pH values and shaken for 24 h at 30 °C. Afterwards, the pH was measured. It can be seen that the pH in general decreases, and the higher the initial pH the higher the pH drop. This is very likely caused by CO_2_ from the atmosphere entering the solution and relatively acidifying the solution. Therefore, the alkalization by the bacteria may be underestimated. The data for this figure can be found in S2 Data.
(EPS)

S10 Fig Six interactions of the 4 bacteria from Fig 1. Two pairs are basically the same (II and III) because the one is the symmetric case of the other, leaving 4 interaction types.
(PNG)

S11 Fig Effect of interaction strength and initial conditions upon simulations. (a) Simulation of *P*. *veronii*–*L*. *plantarum* coculture, in which the proton concentration is kept high and both species can coexist in many cases; (b–d) show simulations like the one in main text Fig 3 but when varying d, the relative strength of the pH changes. d_x_ = y means that the species x changes the proton concentration y times stronger than the other species in coculture. As can be seen, there is no effect on the successive growth and a shift of the outcome for the bistability and the mutual stabilization. The only case in which a qualitative difference can be found is the murder suicide case shown in (a). (e) Simulation of *S*. *marcescens* growth over time at different initial proton concentrations. The lower the initial proton concentration, the longer *S*. *marcescens* survives. The parameters are the ones listed above. (f–i) Simulations of the single species’ growth over time. *L*. *plantarum* and *C*. *ammoniagenes* show an Allee effect; *P*. *veronii* and *S*. *marcescens* show ecological suicide as observed in the experiments. The parameters are the ones listed above. The data for this figure can be found in S2 Data.
(PNG)

S12 Fig Coexistence at the murder suicide case. In the murder suicide case, starting at very low percentages of *P*. *veronii* (lower part of Fig 3C, third panel) leads to oscillatory dynamics that allow coexistence of *L*. *plantarum* and *P*. *veronii* over time.
(EPS)

S13 Fig Effect of periodic dilution on simulation outcomes. The Eqs 1 and 2 were used for this simulation, but every 5 time units, the population densities were diluted by the dilution factor and the proton concentration was set to p ÷ dilutionfactor + (dilutionfactor − 1) ÷ dilutionfactor × po, with “po” the proton concentration of the medium that is used for dilution, which is equal to the proton concentration of the medium the experiment was started in. (A) In the case of the Allee effect, low dilution rates do not qualitatively change the outcome. At high enough dilution rates, the bacteria cannot establish growth anymore, but die out. (B) Also, for ecological suicide, low dilution rates do not change the outcome. Increasing the dilution rates allows for survival because the bacteria are hindered in changing the environment in too detrimental ways. However, at even higher dilution rates, the bacteria are “outdiluted.” Based on this observation, the effect of increased dilution rates upon the interaction cases can be understood. (C) Bistability case consisting of 2 bacteria that show the Allee effect; therefore, increasing the dilution rate too much can kill them and allow none to grow. (D) High dilution rates can kill *L*. *plantarum* (Allee effect) and support *S*. *marcescens* (ecological suicide) and thus allow partial survival of *S*. *marcescens*. (E) Extended suicide stays mostly unaffected by increasing the dilution rate. At dilution factor 5, an area of coexistence can be observed although at low cell densities (insert), which makes it questionable whether this state can be observed in an experimental system at all. (F) *P*. *veronii* and *S*. *marcescens* both show ecological suicide. Therefore, increasing the dilution rates facilitates their survival, which leads at low dilution rates to increased coexistence and high dilution rates allows for survival of the monocultures under conditions that favor the respective species.
(PNG)

S14 Fig Membership functions for different inputs, parameters, and output for the fuzzy rules. (PNG)

S15 Fig Results of fuzzy logic–based simulation. The plots show the survival of A and/or B for different initial fractions of species A and/or species B, and different initial pH values the presence of the species A, B, or both after 200 iterations. The relative strength was set to 0.66 in (A), 2 in (B), 5 in (C, upper), 0.2 in (C, lower), and 1.5 in (D). In A, B, and D, changing the relative strength does not qualitatively change the outcome.
(PNG)

S16 Fig Bistability depends on pH change and is influenced by initial pH value. (A) Coculture of *L*. *plantarum* and *C*. *ammoniagenes* without glucose and urea does not change pH and thus the two species coexist (B–D). The more acidic the initial pH value, the more likely the *C*. *ammoniagenes* (preferring basic conditions) wins. Therefore, the negative fixed point shifts more and more towards higher initial fractions of *L*. *plantarum*. The data for this figure can be found in S2 Data.
(PNG)

S17 Fig Successive growth. *S*. *marcescens* cannot promote growth of *L*. *plantarum* when pH change is hindered. Absence of glucose and urea (A) or buffer (B) stops *S*. *marcescens* from changing the pH and thus does lead to the extinction of *L*. *plantarum* even in the presence of *S*. *marcescens*. The data for this figure can be found in S2 Data.
(PNG)

S18 Fig Murder suicide. In base with 10 g/L glucose and 100 mM phosphate. *L*. *plantarum* can grow and acidify the medium. *P*. *veronii* is not sufficiently acidifying the medium to kill itself. The data for this figure can be found in S2 Data.
(PNG)

S1 Table Fuzzy logic–based rules for change in cell density. pH is current pH, and “cell_density_change” is the change of the cell density. “IF pH ['low'] AND preferred_pH ['low'] THEN cell_density_change ['positive']” means: if pH is low and preferred pH is low, then the change of cell density is positive.
(DOCX)

S2 Table Fuzzy logic–based rules for change in pH. pH_change_ability is the direction of the pH change for the specific species. It was always set to −1 or 1.
(DOCX)

S1 Text Description, discussion, and analysis of fuzzy logic and differential equation models. (DOCX)

S1 Data Data for Figs 1, 2 and 4. (XLSX)

S2 Data Data for S2, S4, S5, S6, S7, S8, S9, S11, S15, S16 and S17 Figs. (ODS)

S3 Data Alignment for phylogenetic tree in S1 Fig. (FASTA)

S4 Data Python code for simulations in Fig 3 and S2, S11, S12, S13 and S15 Figs. (IPYNB)

## Abbreviations

CFU: colony-forming unit
OD: optical density
PBS: phosphate-buffered saline
