# Supplementary figures and images for: Modifying and reacting to the environmental pH can drive bacterial interactions
Source: PLoS Biol. 2018 Mar 14;16(3):e2004248. doi: 10.1371/journal.pbio.2004248 (PMC5868856; doi:10.1371/journal.pbio.2004248)

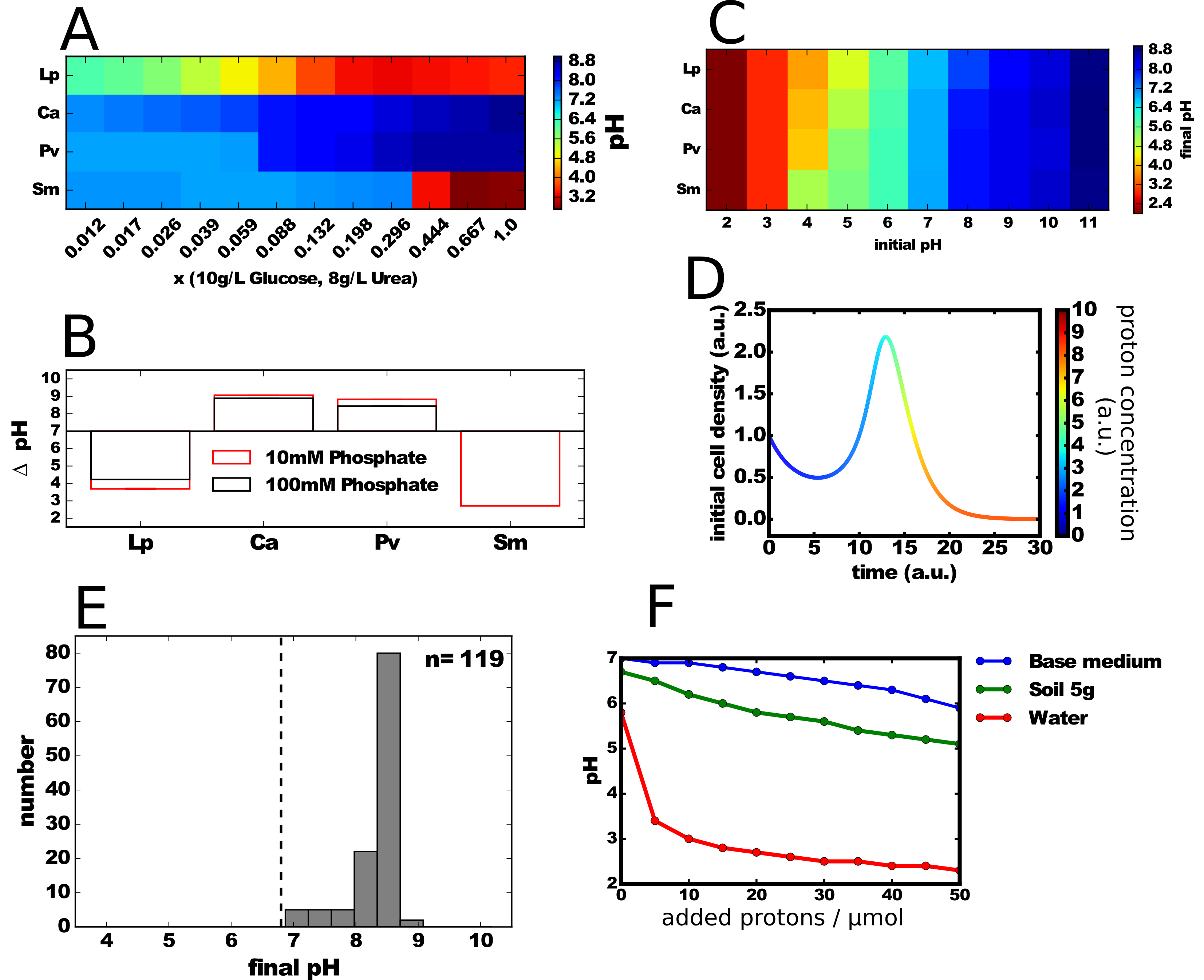

Supplement: S2 Fig — (A) The higher the nutrient concentration, the stronger the pH change. (B) Buffer hinders the pH change. In the case of S. marcescens, there is no pH shift at all at high-buffer concentrations. Base buffer with 10 g/L glucose and 8 g/L urea. Bacteria were grown in the media for 24 h. Error bars show SEM but can barely be seen. In (A), 10 mM phosphate buffer was used. (C) The four species were grown in base, 100 mM phosphate with different initial pH values to test how their growth rate depends on the pH (Fig 1B). The absence of glucose and urea and the presence of buffer lead to rather small pH changes in growth. However, the high starting pH values drop significantly over time, which is likely caused by dissolution of CO2 in the growth medium (see S7 Fig). (D) Simulation of microbial growth illustrates how pH changes can lead to death of the population. The simulation was done as described in the main text and in the Supporting information below. The ppref is set to 3.5. The initial proton concentration is set to 1. The bacteria increase the proton concentrations. In this way, they first facilitate their own growth but then keep on changing the proton concentration and kill themselves. (E) The soil bacteria from main text Fig 1A also show a pH change in Luria-Bertani medium. Again, the pH was measured after 24 h of growth of the soil bacteria in the medium. (F) The buffering capacity of the soil that the strains in Fig 1A were isolated from is lower than that of base medium, which means that pH effects in soil may be even more pronounced than in that medium. The data for this figure can be found in S2 Data. ppref, preferred proton concentration. (PNG) [file pbio.2004248.s002.png]

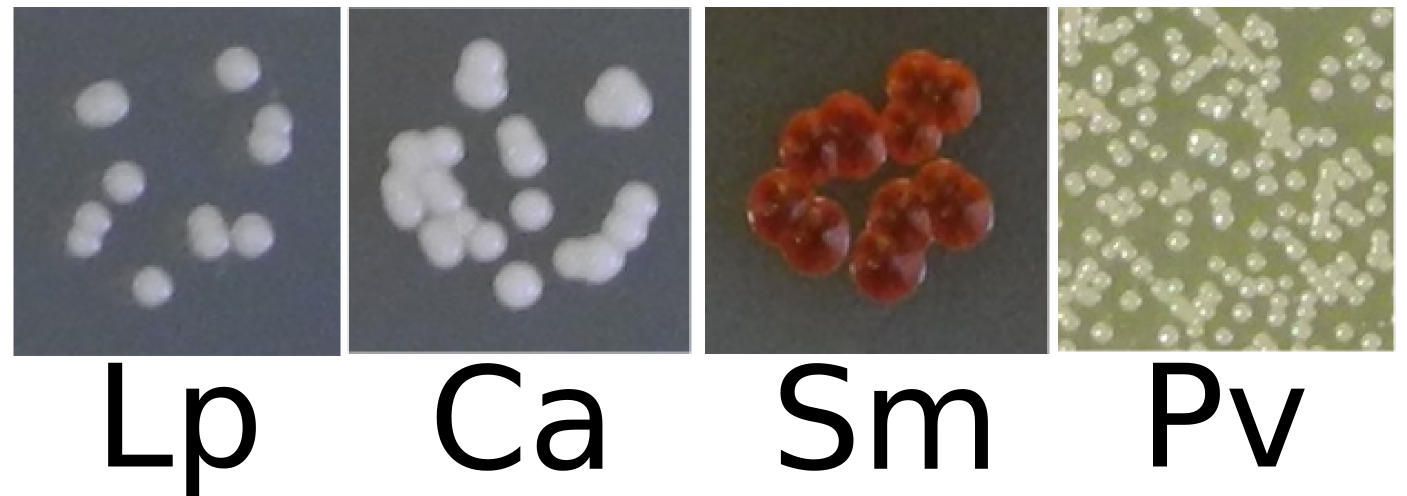

Supplement: S3 Fig — The colony color and morphology allow us to distinguish them pretty well, with the exception of L. plantarum and C. ammoniagenes. Although L. plantarum can be recognized because it is more translucent than C. ammoniagenes, C. ammoniagenes and L. plantarum were distinguished by plating them on agar plates with pH 4 and 10, respectively. L. plantarum only grows on the pH 4 plates and C. ammoniagenes only on the pH 10 plates. (PNG) [file pbio.2004248.s003.png]

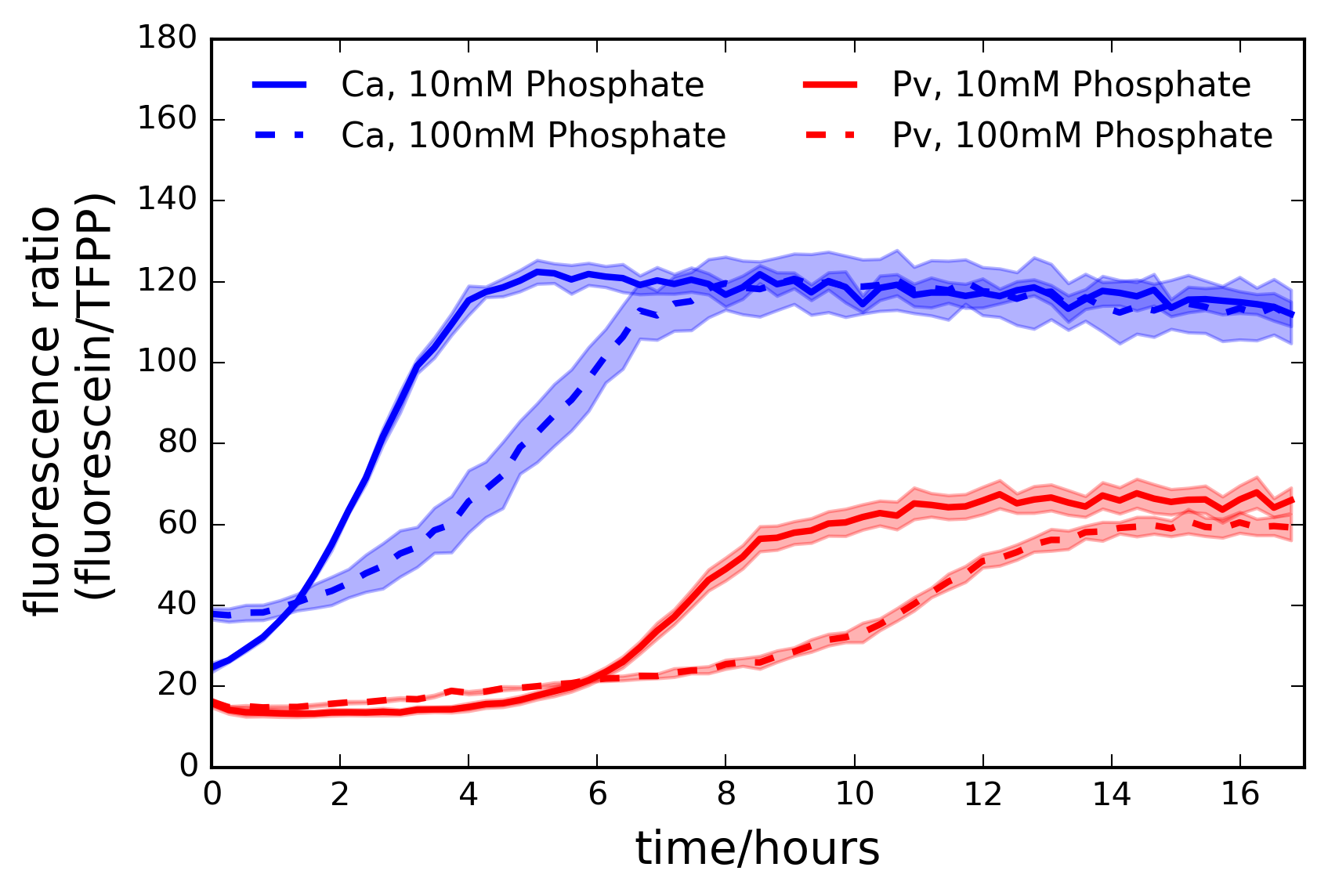

Supplement: S4 Fig — C. ammoniagenes and P. veronii were grown in base medium, 10 g/L glucose, 8 g/L urea, and with 10 or 100 mM phosphate, respectively. The change of the pH was followed in real time by measuring the ratio of the pH-dependent fluorophore fluorescein and the pH-independent fluorophore TFPP. Both dyes were enclosed in nanobeads as described in [5]. The earlier the pH change happens, the longer the bacteria are exposed to the—for them—either benign (C. ammoniagenes) or detrimental (P. veronii) environment, which either increases or decreases their fitness as shown in Fig 2. The lines show the mean of 8 replicas, and the shaded region shows SEM. The data for this figure can be found in S2 Data. TFPP, tetrakis(pentafluorophenyl) porphyrin. (PNG) [file pbio.2004248.s004.png]

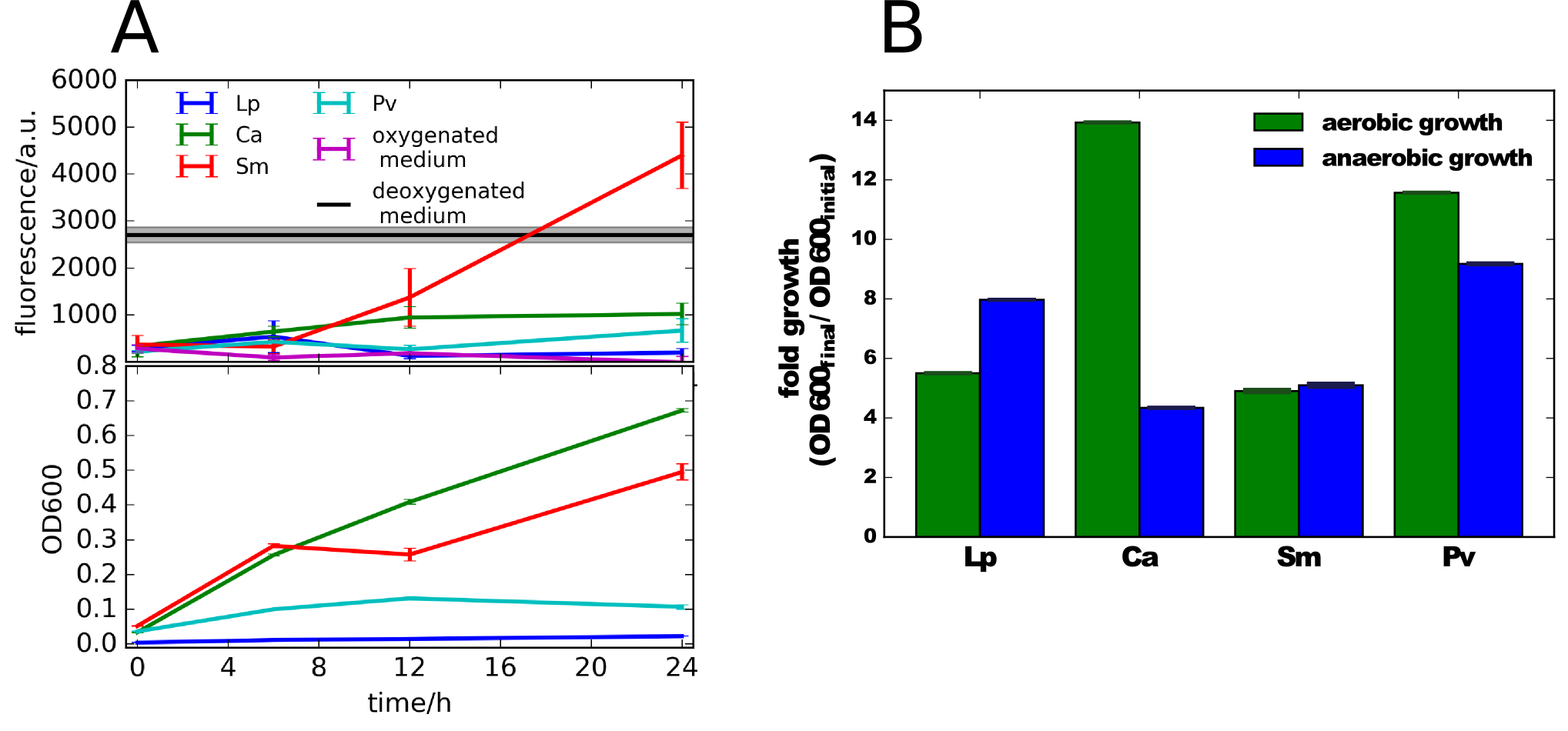

Supplement: S5 Fig — (A) Bacteria were grown in base medium with 100 mM phosphate buffer, pH 7 supplemented with 10 g/L glucose and 8g/L urea, at 30 °C in 200 μL volumes in deepwell plates with 1,350 rpm shaking and thus just like the samples for the other experiments. The oxygen content was measured with the extracellular oxygen consumption assay (ab197243; Abcam, Cambridge, MA). The higher the fluorescence signal, the less oxygen is in the sample. Oxygenated medium is just the medium without bacteria but otherwise treated like the cultures; deoxygenated medium is medium with 2 M sodium sulfite, which reacts with oxygen. As can be seen, oxygen depletion by the bacteria is moderate by most bacteria, with the exception of S. marcescens, which produces a rather strong anaerobic environment. This depletion of oxygen goes in line with a rather high cell density. S. marcescens was used in the experiments for successive growth with L. plantarum and stabilization with P. veronii. S. marcescens may support L. plantarum not only by lowering the pH but also depleting O2. Moreover, this oxygen depletion may also destabilize the proposed coexistence between S. marcescens and P. veronii and thus is a possible reason that we had difficulties finding it experimentally. The strong error for the OD of S. marcescens is likely caused by clumping of the cells, which disturbs the OD measurement. (B) The bacteria were grown for 24 h in base medium with 100 mM phosphate buffer and 10 g/L glucose and 8g/L urea at RT in 200 μL in deep-well plates either with normal aeration or in an anaerobic chamber containing 5% hydrogen, 20% carbon dioxide, and 75% nitrogen as atmosphere. The OD600 was measured at the beginning and end of the experiment to obtain the fold growth of the bacteria. The influence of oxygen on the growth is surprisingly small, with only C. ammoniagenes being strongly inhibited by the absence of oxygen. L. plantarum is an anaerobic bacterium and thus profits from the absence of oxygen. The data fo [file pbio.2004248.s005.png]

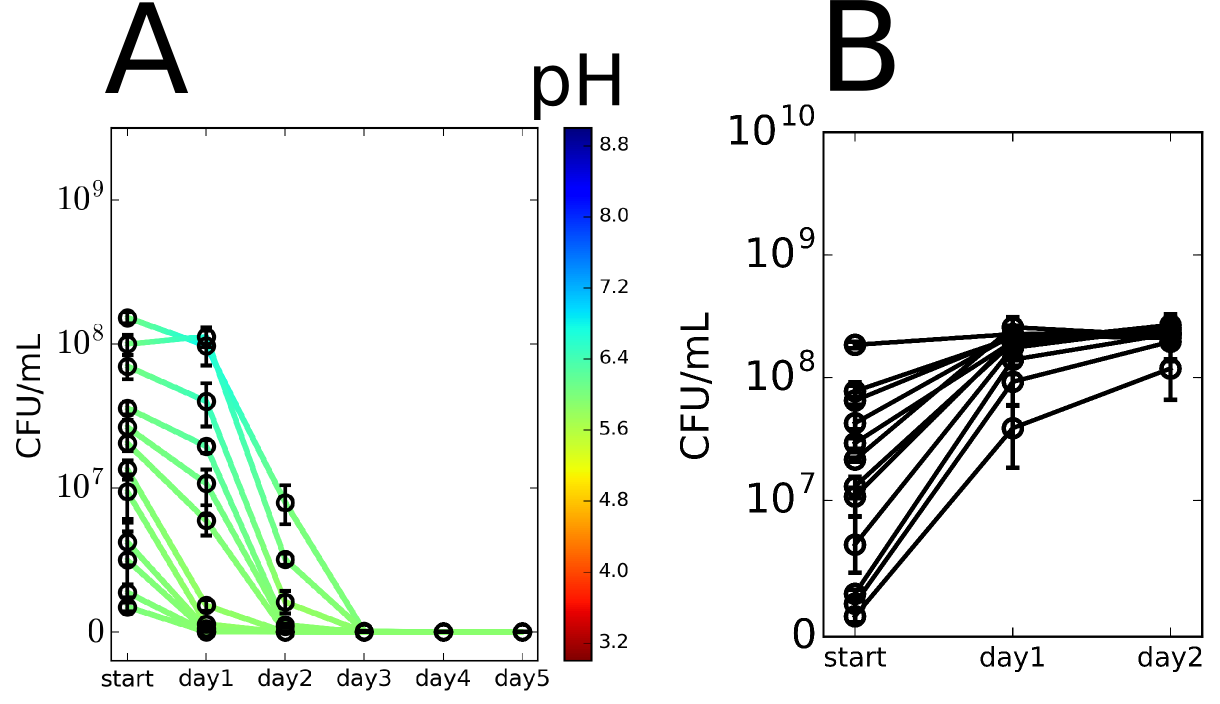

Supplement: S6 Fig — (A) C. ammoniagenes in base buffer without additional urea or glucose and a 1/10x dilution every 24 h. Starting at pH 6, the bacteria cannot sufficiently change the pH and thus go extinct. (B) At the same conditions but a starting pH of 7, all populations survive independent of the initial cell density (for B, the pH change was not recorded). The data for this figure can be found in S2 Data. (PNG) [file pbio.2004248.s006.png]

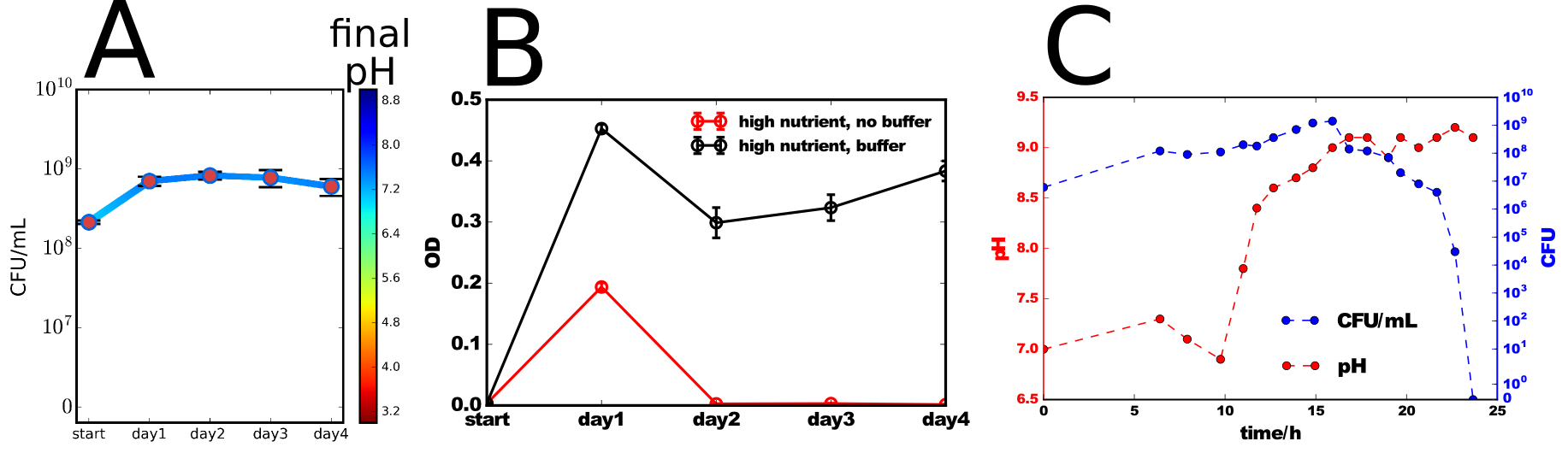

Supplement: S7 Fig — (A) In the absence of glucose and urea, P. veronii is not able to increase the pH drastically and thus does not kill itself. However, there is a small increase in pH, which is likely caused by the fact that the base medium contains peptides as carbon sources. In this case, excess ammonia is excreted and increases the pH (see S1 Fig). Daily dilution of 1/100x. (B) Under daily batch culture with dilution (1/100x), the OD at high-nutrient concentrations first increases and then drops to zero. In contrast, the CFU drops to zero after the first day of growth as a result of ecological suicide (Fig 2b). The OD is measuring light scattering and thus the presence of cells regardless of whether they are dead or alive. However, CFU just measures the living cells that are able to form colonies when plated on agar. Therefore, after the first day, we have zero living but a high number of dead cells. This shows that the bacteria could initially grow but later died out. Increased buffering of the media allows the population to survive multiple growth-dilution cycles, and the OD remains high (Fig 2b). (C) Ecological suicide of P. veronii can be followed with high temporal resolution, such that initially the CFU increases, but as the pH increases, the CFU starts to drop until the population goes extinct. Initially, the CFU increases, but as the pH increases, they start to drop till they go extinct completely. This phenomenon will be discussed in more detail in a separate publication. The data for this figure can be found in S2 Data. CFU, colony-forming unit; OD, optical density. (PNG) [file pbio.2004248.s007.png]

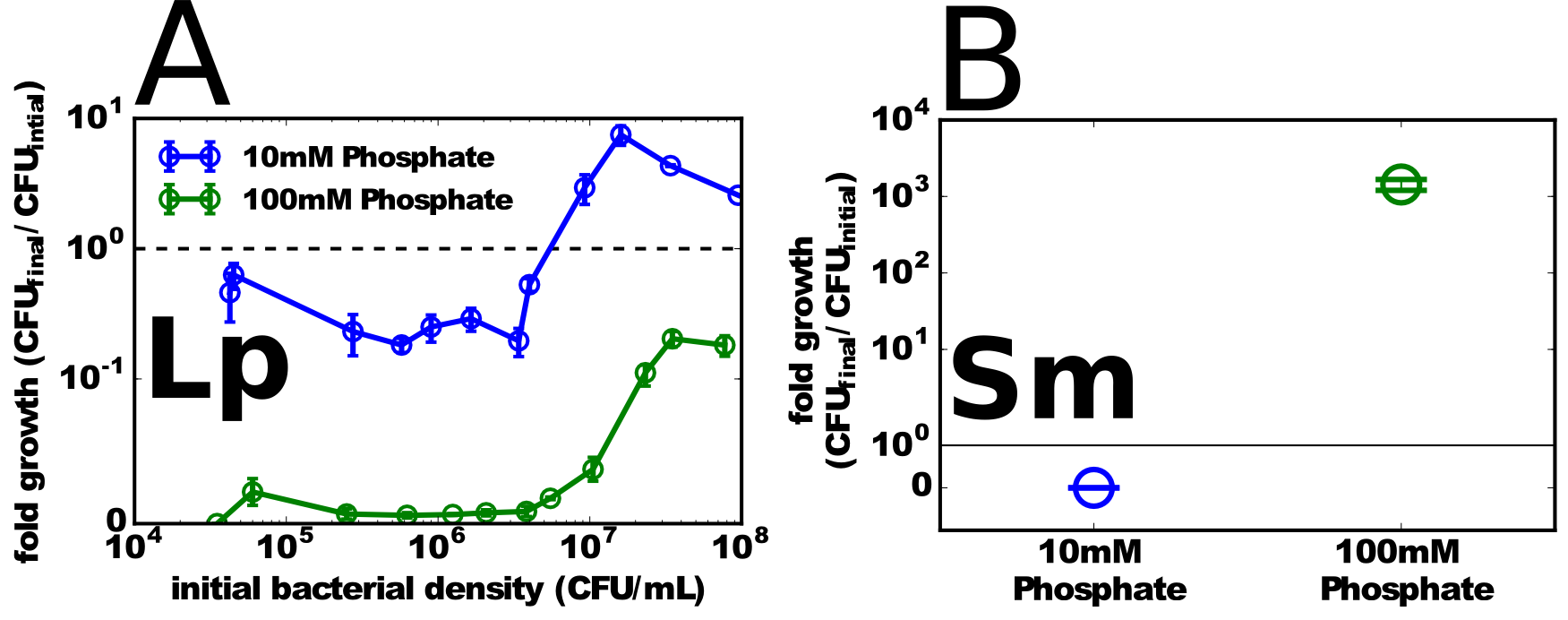

Supplement: S8 Fig — (A) Starting at a high pH of 10.2, L. plantarum also shows a density-dependent fitness in media containing urea and glucose. Estimating the fold growth after 24 h only at initial high cell densities, a net growth could be achieved; at low cell densities, the bacteria die. Adding more buffer and thus making pH change more difficult leads to death at all initial cell densities. (B) S. marcescens strongly acidifies the environment and thus causes cell death (again the fold growth within 24 h was measured), but in this case, the addition of buffer leads to positive net growth—which shows that suicide is indeed caused by the pH change. The data for this figure can be found in S2 Data. (PNG) [file pbio.2004248.s008.png]

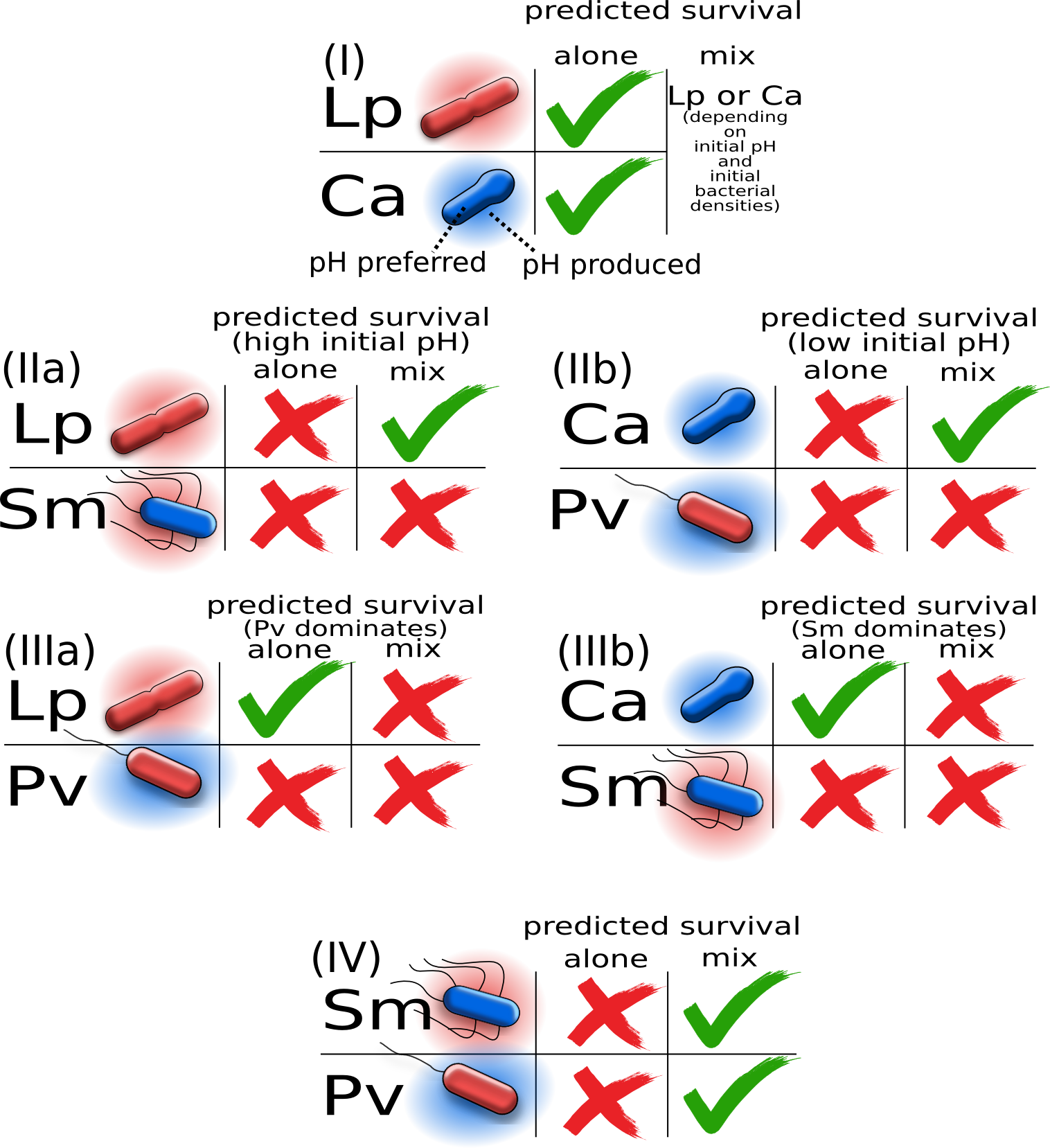

Supplement: S10 Fig — Two pairs are basically the same (II and III) because the one is the symmetric case of the other, leaving 4 interaction types. (PNG) [file pbio.2004248.s010.png]

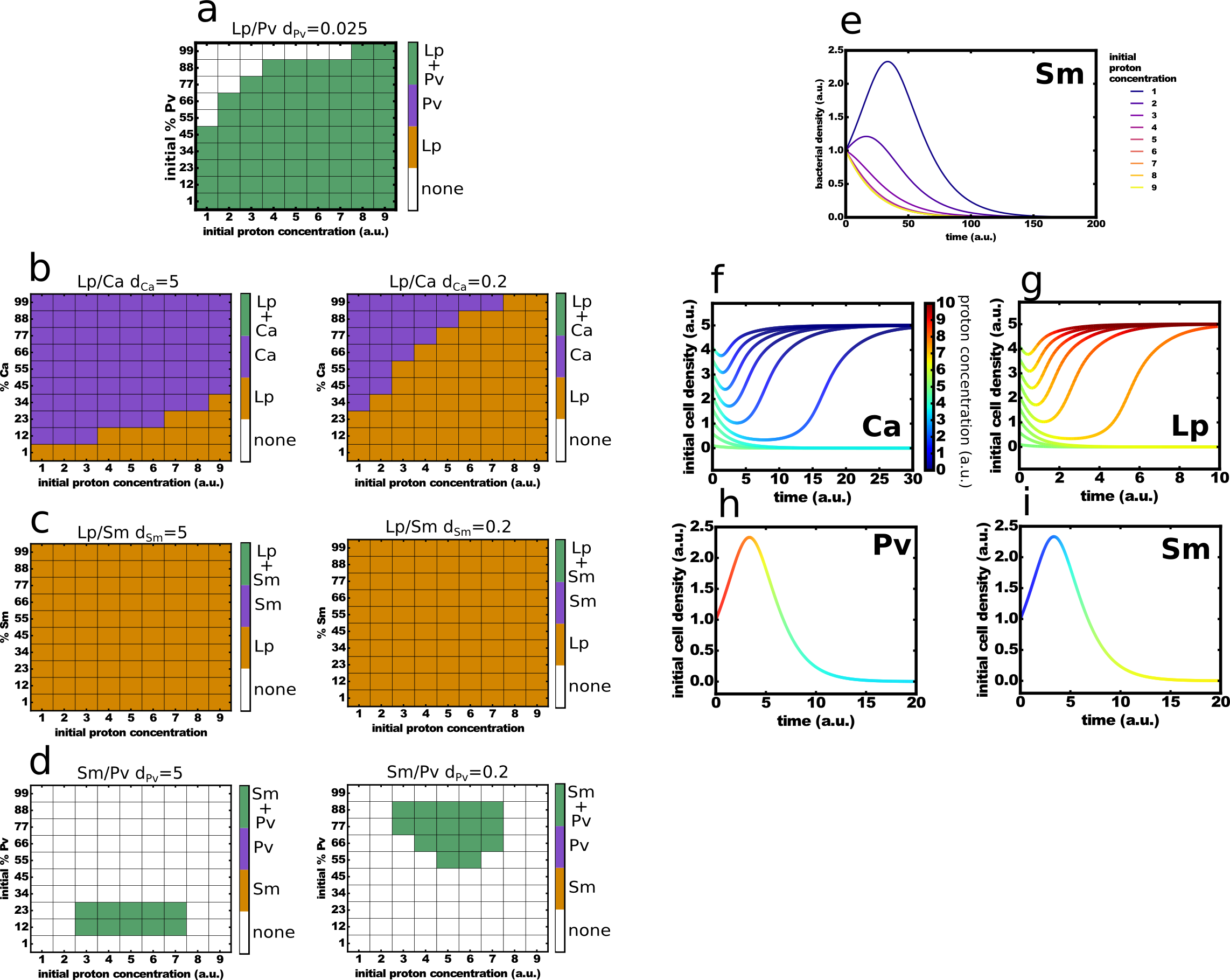

Supplement: S11 Fig — (a) Simulation of P. veronii–L. plantarum coculture, in which the proton concentration is kept high and both species can coexist in many cases; (b–d) show simulations like the one in main text Fig 3 but when varying d, the relative strength of the pH changes. dx = y means that the species x changes the proton concentration y times stronger than the other species in coculture. As can be seen, there is no effect on the successive growth and a shift of the outcome for the bistability and the mutual stabilization. The only case in which a qualitative difference can be found is the murder suicide case shown in (a). (e) Simulation of S. marcescens growth over time at different initial proton concentrations. The lower the initial proton concentration, the longer S. marcescens survives. The parameters are the ones listed above. (f–i) Simulations of the single species’ growth over time. L. plantarum and C. ammoniagenes show an Allee effect; P. veronii and S. marcescens show ecological suicide as observed in the experiments. The parameters are the ones listed above. The data for this figure can be found in S2 Data. (PNG) [file pbio.2004248.s011.png]

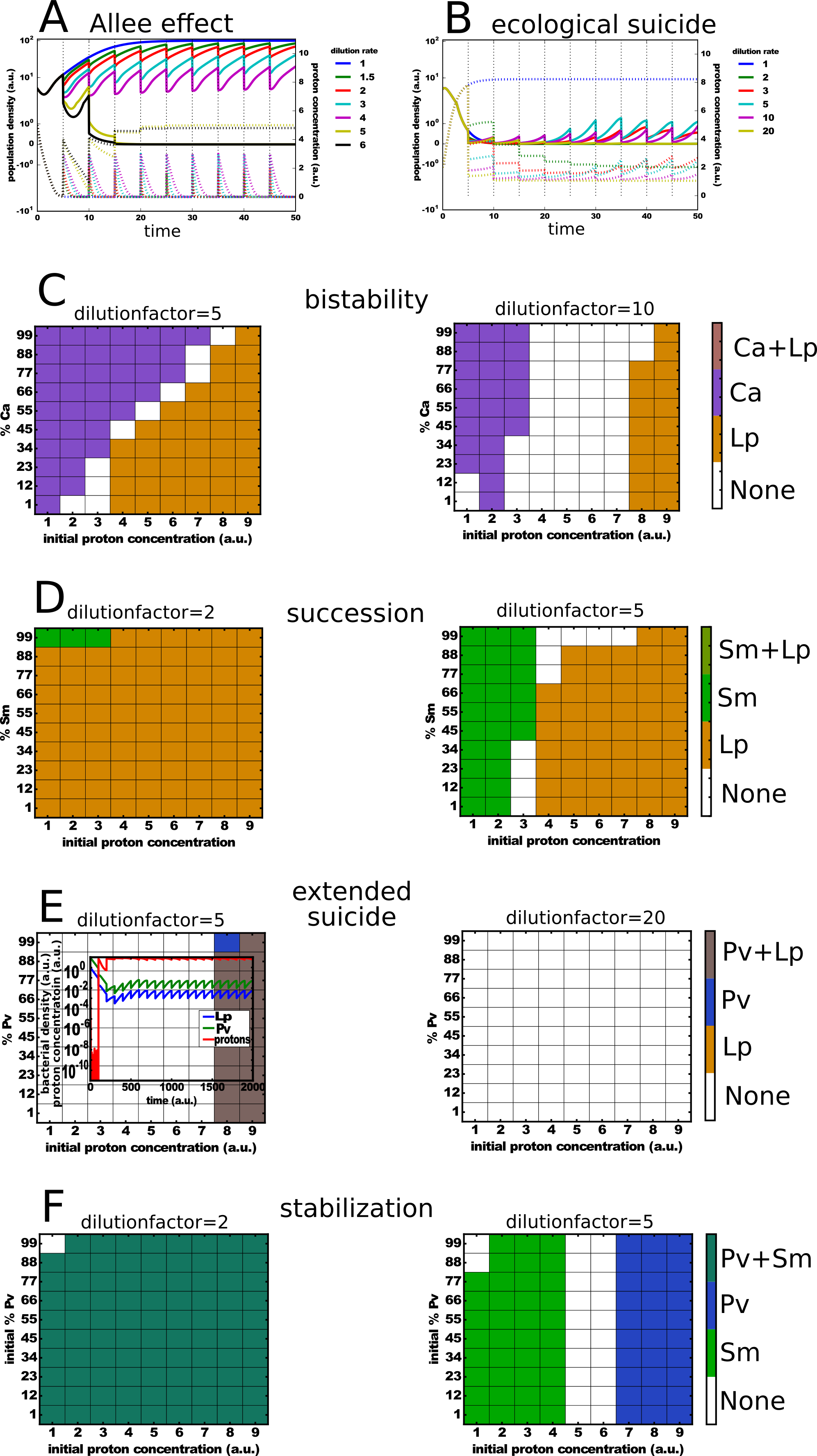

Supplement: S13 Fig — The Eqs 1 and 2 were used for this simulation, but every 5 time units, the population densities were diluted by the dilution factor and the proton concentration was set to p ÷ dilutionfactor + (dilutionfactor − 1) ÷ dilutionfactor × po, with “po” the proton concentration of the medium that is used for dilution, which is equal to the proton concentration of the medium the experiment was started in. (A) In the case of the Allee effect, low dilution rates do not qualitatively change the outcome. At high enough dilution rates, the bacteria cannot establish growth anymore, but die out. (B) Also, for ecological suicide, low dilution rates do not change the outcome. Increasing the dilution rates allows for survival because the bacteria are hindered in changing the environment in too detrimental ways. However, at even higher dilution rates, the bacteria are “outdiluted.” Based on this observation, the effect of increased dilution rates upon the interaction cases can be understood. (C) Bistability case consisting of 2 bacteria that show the Allee effect; therefore, increasing the dilution rate too much can kill them and allow none to grow. (D) High dilution rates can kill L. plantarum (Allee effect) and support S. marcescens (ecological suicide) and thus allow partial survival of S. marcescens. (E) Extended suicide stays mostly unaffected by increasing the dilution rate. At dilution factor 5, an area of coexistence can be observed although at low cell densities (insert), which makes it questionable whether this state can be observed in an experimental system at all. (F) P. veronii and S. marcescens both show ecological suicide. Therefore, increasing the dilution rates facilitates their survival, which leads at low dilution rates to increased coexistence and high dilution rates allows for survival of the monocultures under conditions that favor the respective species. (PNG) [file pbio.2004248.s013.png]

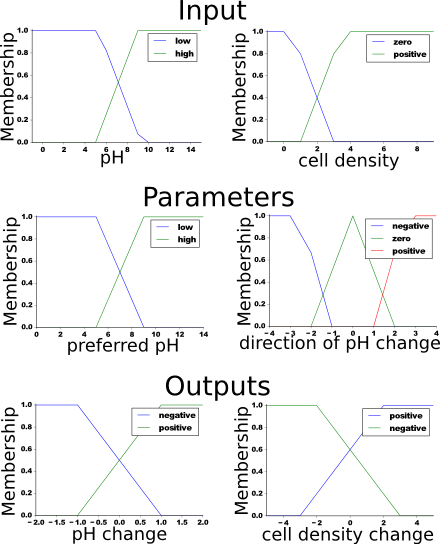

Supplement: S14 Fig — (PNG) [file pbio.2004248.s014.png]

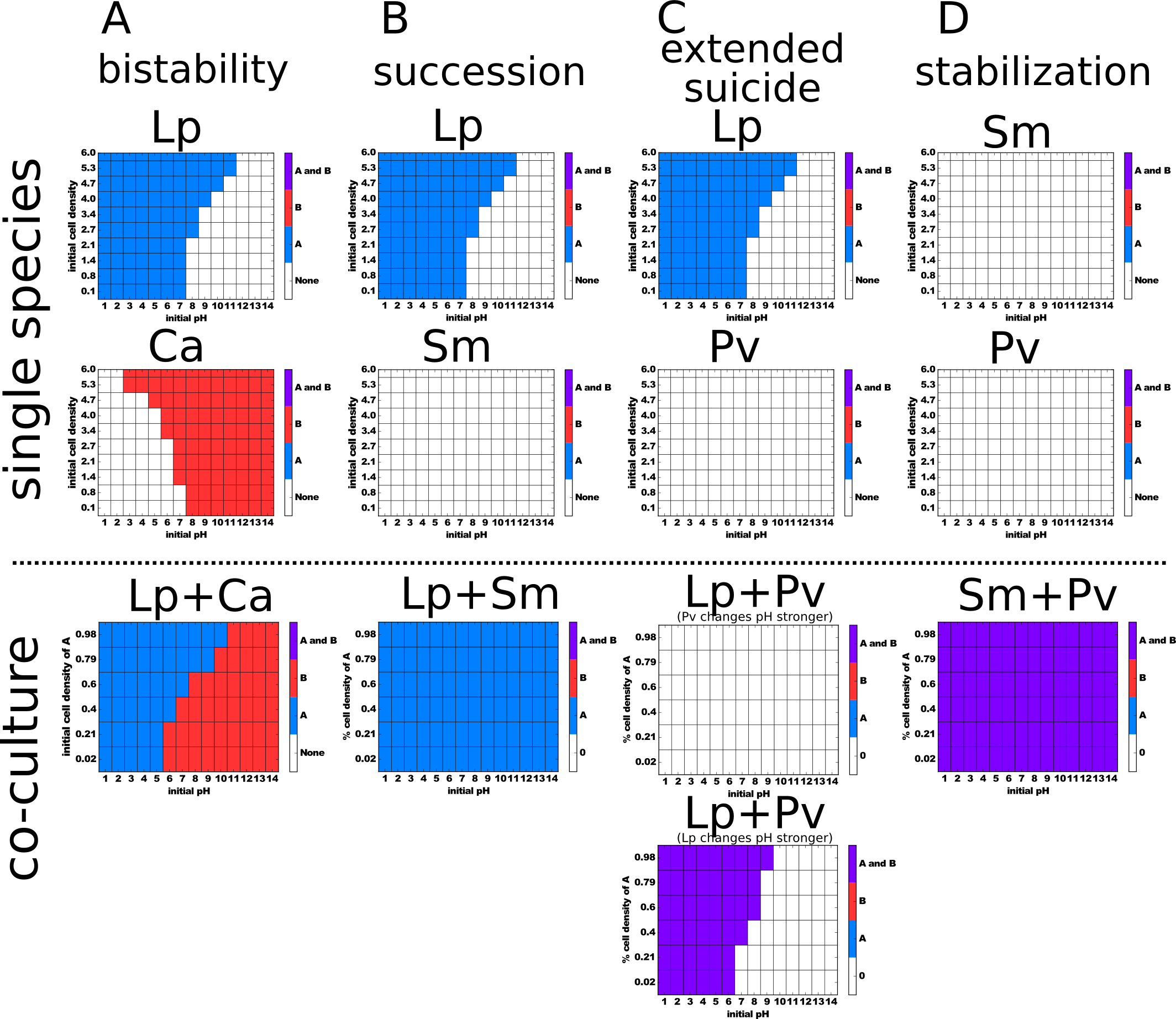

Supplement: S15 Fig — The plots show the survival of A and/or B for different initial fractions of species A and/or species B, and different initial pH values the presence of the species A, B, or both after 200 iterations. The relative strength was set to 0.66 in (A), 2 in (B), 5 in (C, upper), 0.2 in (C, lower), and 1.5 in (D). In A, B, and D, changing the relative strength does not qualitatively change the outcome. (PNG) [file pbio.2004248.s015.png]

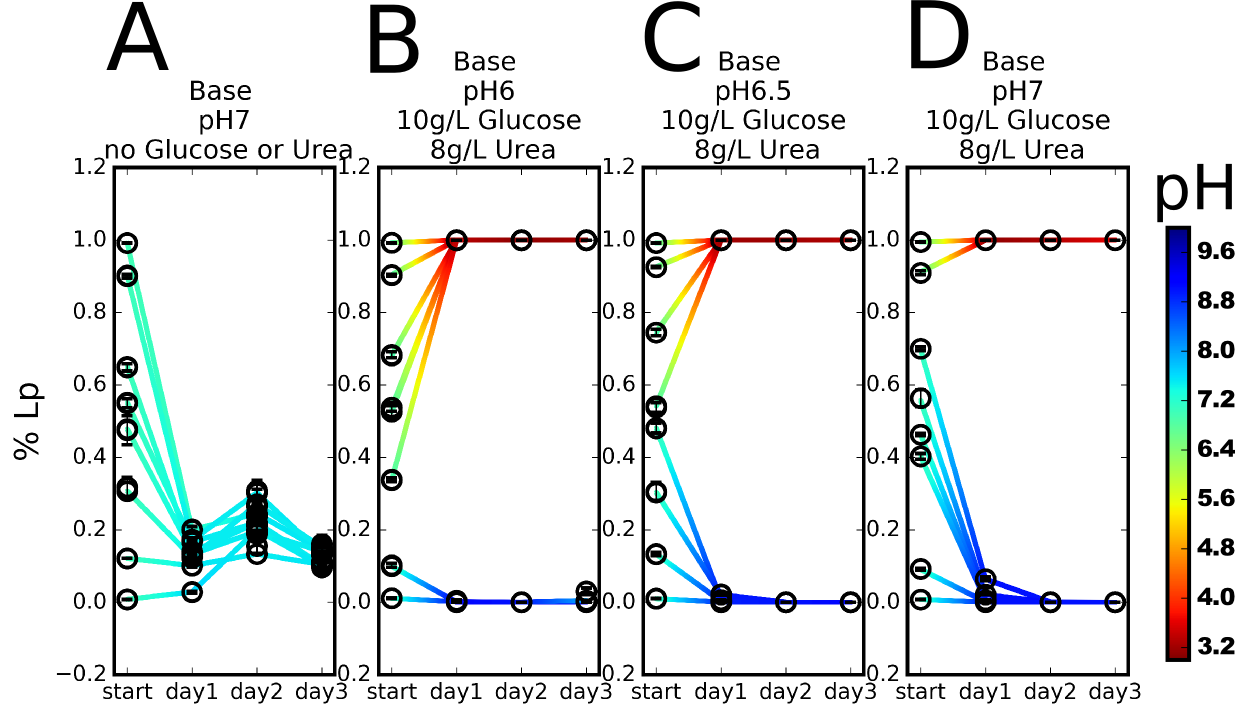

Supplement: S16 Fig — (A) Coculture of L. plantarum and C. ammoniagenes without glucose and urea does not change pH and thus the two species coexist (B–D). The more acidic the initial pH value, the more likely the C. ammoniagenes (preferring basic conditions) wins. Therefore, the negative fixed point shifts more and more towards higher initial fractions of L. plantarum. The data for this figure can be found in S2 Data. (PNG) [file pbio.2004248.s016.png]

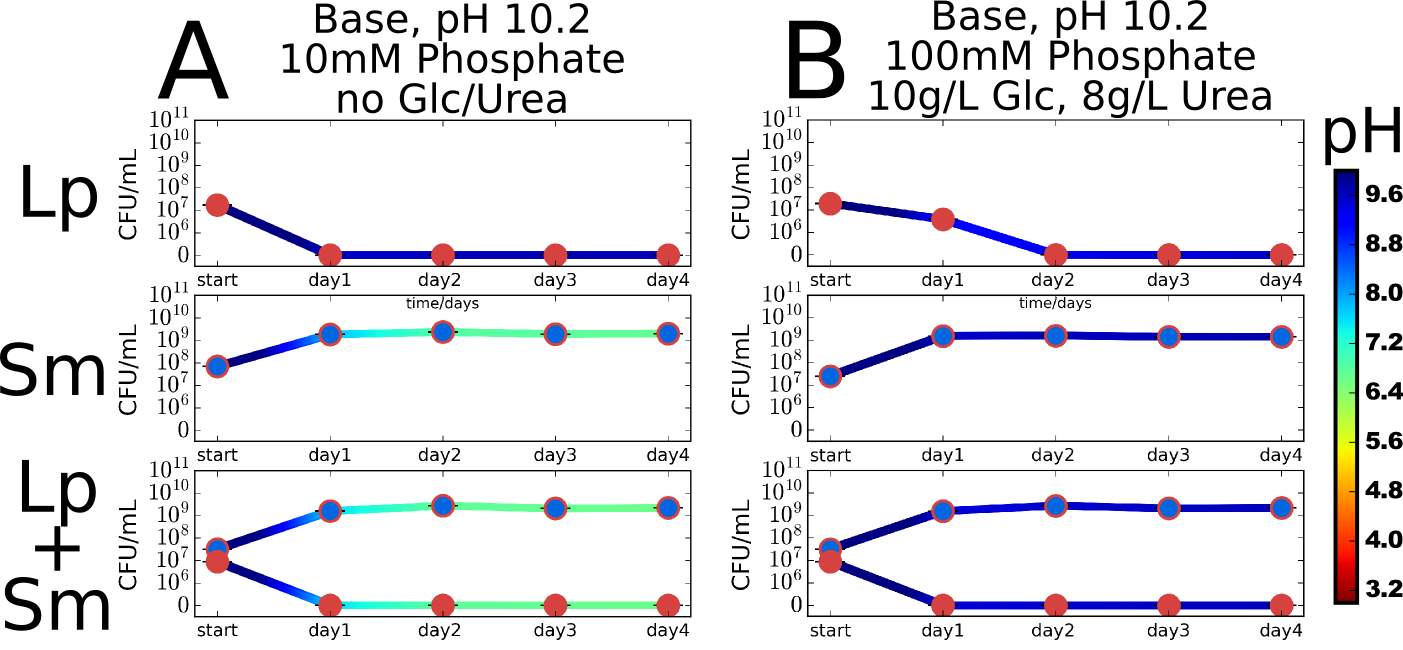

Supplement: S17 Fig — S. marcescens cannot promote growth of L. plantarum when pH change is hindered. Absence of glucose and urea (A) or buffer (B) stops S. marcescens from changing the pH and thus does lead to the extinction of L. plantarum even in the presence of S. marcescens. The data for this figure can be found in S2 Data. (PNG) [file pbio.2004248.s017.png]

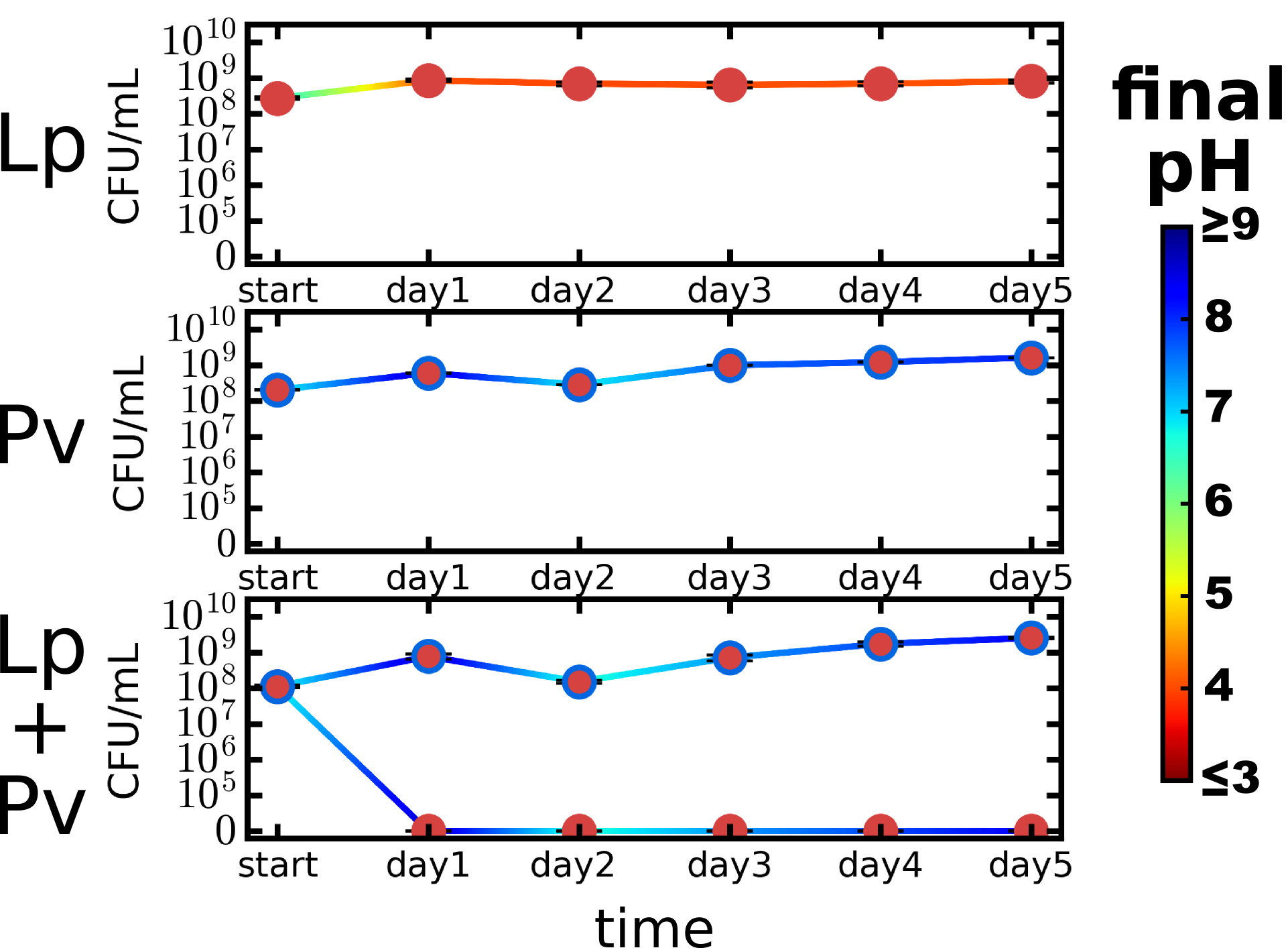

Supplement: S18 Fig — In base with 10 g/L glucose and 100 mM phosphate. L. plantarum can grow and acidify the medium. P. veronii is not sufficiently acidifying the medium to kill itself. The data for this figure can be found in S2 Data. (PNG) [file pbio.2004248.s018.png]
