# Supplementary material for: Modifying and reacting to the environmental pH can drive bacterial interactions
Source: PLoS Biol. 2018 Mar 14;16(3):e2004248. doi: 10.1371/journal.pbio.2004248 (PMC5868856; doi:10.1371/journal.pbio.2004248)
Supplement: S1 Table — pH is current pH, and “cell_density_change” is the change of the cell density. “IF pH ['low'] AND preferred_pH ['low'] THEN cell_density_change ['positive']” means: if pH is low and preferred pH is low, then the change of cell density is positive. (DOCX) [file pbio.2004248.s019.docx]

| Rule 1 | IF (pH['low'] AND preferred_pH['low']) OR (pH['high'] AND preferred_pH['high'])  THEN cell_density_change['positive'] |
| --- | --- |
| Rule 2 | IF (pH['low'] AND preferred_pH['high']) OR (pH['high'] AND preferred_pH['low'])  THEN cell_density_change['negative']) |
