# Supplementary material for: Modifying and reacting to the environmental pH can drive bacterial interactions
Source: PLoS Biol. 2018 Mar 14;16(3):e2004248. doi: 10.1371/journal.pbio.2004248 (PMC5868856; doi:10.1371/journal.pbio.2004248)
Supplement: S2 Table — pH_change_ability is the direction of the pH change for the specific species. It was always set to −1 or 1. (DOCX) [file pbio.2004248.s020.docx]

| Rule 3 | IF cell_density['positive'] AND pH_change['positive'] THAN pH_change['positive]) |
| --- | --- |
| Rule 4 | IF cell_density['positive'] AND pH_change['negative'] THAN pH_change['negative]) |
| Rule 5 | IF cell_density['zero'] THAN pH_change['zero']) |
