# Supplementary material for: Modifying and reacting to the environmental pH can drive bacterial interactions
Source: PLoS Biol. 2018 Mar 14;16(3):e2004248. doi: 10.1371/journal.pbio.2004248 (PMC5868856; doi:10.1371/journal.pbio.2004248)
Supplement: S1 Text — (DOCX) [file pbio.2004248.s021.docx]

**Differential equation based model**

The time evolution of the bacterial cell density and the proton concentration in the the cultured bacteria either as single species or pairs has been modeled by two coupled differential equations.

$\frac{\partial n_{a/b}}{\partial t}=n_{a/b}\left( 1-\frac{n_{a/b}}{K} \right)\left( e^{\frac{-\left( p-p_{pref} \right)^{2}}{\sigma^{2}}}-\delta\right)$ **(1)**

$\frac{\partial p}{\partial t}=\left( c_{a}n_{a}+c_{b}n_{b}d \right)\left( \frac{p}{b}\left( 2b-p \right) \right)$ **(2)**

,where n_a/b_ are the cell densities of species A and B respectively, K is the carrying capacity, p is the proton concentration, p_pref_ is the preferred proton concentration c_a/b_ are the proton production rates for species A and B. σ was set to 4 and δ to 0.5. The first equation basically is a logistic growth of the bacteria multiplied with Gaussian function that ensures that the growth rate becomes maximal when the proton concentration equals the preferred proton concentration. For proton concentrations far away from the preferred value this factor becomes negative and thus the bacteria die. The proton concentration is treated phenomenologically, and changes according to the proton production rate of the cells c_a/b_ and the cell density n_a/b_ of each type. The second term in equation (2) ensures that the proton concentration stays within [0,2b], whereas b was set to 5 in all cases. Thus the proton concentration can in principle vary between 0 and 10. This takes into account that bacteria can not change the proton concentration forever, but they saturate. The saturation pH is set by how far they can secrete/remove protons into/from the environment and thus how far they can act against the increasing difference in chemical potential between inside and outside the cell. However the exact shift of the pH depends of course on the species and the amount and type of accessible nutrient (see Fig. 1 and Supplementary Fig. 2). We chose 1 and 9 as p_pref_ for species that prefer high or low proton concentration. However, for (d) coexistence could only be achieved if p_pref_  were not chosen to extreme. C_a/b_ was chosen to be +/-0.1, which means that the rate by which the bacteria change the proton concentration is 1/10 of their growth rate. By tuning d the relative strength of the change in proton concentration by the two species can be changed. Mostly d is set to 0.8 to avoid total symmetry between the two species, which might make the system stuck in unstable fixed points. However, it was set close to 1 in most cases, except (c) where the relative strength in interaction of the both species leads to qualitatively different outcomes as shown in Supplementary Fig. 11.

The carrying capacity was set to 100 for all the phase diagrams in Fig. 3, to allow for sufficient bacterial growth but at the same avoid too long simulation times. The phase diagrams in the fist three rows in Fig. 3 show the outcome of the simulation after t=500 a.u.. A final values of lower than 10^-5^ was counted as no survival, otherwise as survival of the species. The above formulas were numerical integrated with the odeint package of Python/Scipy.

The parameters for the phase diagrams in main text Fig. 3 (upper three panels) are:

(a) p_pref Lp_= 9, p_pref Ca_= 1, cp_Lp_= 0.1, cp_Ca_ = -0.1, d_Ca_=0.8

(b) p_pref Lp_= 9, p_pref Sm_= 1, cp_Lp_= 0.1, cp_Sm_ = 0.1, d_Sm_=0.8

(c) p_pref Lp_= 9, p_pref Pv_ = 9, cp_Lp_= 0.1, cp_Pv_ = -0.1, d_Pv_=40

(d) p_pref Sm_=3 , p_pref Pv_ = 7, cp_Sm_= 0.1, cp_Pv_ = -0.1, d_Sm_=0.8

for (d) the p_pref_ of the both species have to be sufficiently close to allow mutual stabilization, which again highlights that also this situation may exist it may be difficult to experimentally find it.

The code for the simulations is provided as S4_Data.

**Normalized equations**

Equations (1) and (2) can be rescaled by setting $n=\frac{n}{K}$, $p=p/\sigma$, $p_{prefa/b}={p_{prefa/b}}/\sigma$, $\alpha=\frac{c_{a}}{c_{b}d}$, $\beta=\frac{Kc_{b}d\sigma}{b}$and $\gamma=2b/\sigma$, which leaves α, β, γ, δ and $p_{pref}$ as the only free parameters:

$\frac{\partial n_{a/b}}{\partial t}=n_{a/b}\left( 1-n_{a/b} \right)\left( e^{-\left( p-p_{prefa/b} \right)^{2}}-\delta\right)$ **(3)**

$\frac{\partial p}{\partial t}=\left( \alpha n_{a}+n_{b} \right)\left( \beta p\left( \gamma-p \right) \right)$ **(4)**

**Stability analysis**

For this equation system the fixed points can be obtained as:

1) n_a_=0, n_b_=0

2) n_a_=0, n_b_=1, p=0

3) n_a_=0, n_b_=1, p=

4) n_a_=1, n_b_=0, p=0

5) n_a_=1, n_b_=0, p=

6) n_a_=1, n_b_=1, p=0

7) n_a_=1, n_b_=1, p=

8+9) n_a_=-1/, n_b_=1, p=p_prefa_+/-sqrt(-ln )/2

10+11) n_a_=1, n_b_=-, p=p_prefb_+/-sqrt(-ln )/2

In the cases 1)-7) the systems has its proton concentration at its extremes (0 or gamma) and also the bacterial densities reach their extremes (0 or 1). 1) is reached in the experiments upon murder suicide where both species go extinct, successive growth and bistability correspond to the fixed points of the type 2) to 5) where one species reaches carrying capacity and the other goes extinct. In all those cases the equation (3) is set to zero by its first two factors, the third factor does not matter.

The cases 8) and 9) correspond to the mutual stabilization case where both species can survive and grow, the proton concentration causes the growth of one of the species to reach zero (eg where the last factor in (3) becomes zero).

To estimate the stability at those fixed points the Eigenvalues of the underlying Jacobian Matrix have been determined and are shown in the following:

1)

2)

3)

4)

5)

6)

7)

In the following we verbalize the obtained eigenvalues and explain their meaning for better understanding. We call the third factor in Eq. 3 growth vs proton concentration.

The stability of the first 7 fixed points (whereas again 1) corresponds to murder suicide, and 3) to 5) to succession and bistability) is mainly set by the choice of the parameters , and and which values the growth vs proton concentration curve (third factor in equation (3)) reaches at the fixed point. Especially note from the definition of , and that is always positive, can only become negative when c_a_ or c_b_ (but not both) are negative and just becomes negative when c_b_ becomes negative. That means the stability of the fixed point is largely set by the value of the growth vs p curve at the fixed point and in which way the bacteria change the proton concentration.

For the single fixed points the conditions are as follows:

1) becomes stable for or and or (for 0>>1). In other words the proton concentration has be far enough away from the optimum that both species show negative growth vs proton concentration values. This way the bacterial densities of both species tend to zero. This fixed point thus corresponds to a situation where the proton concentration is that extreme that both species die out, this fixed point is reached in the extended suicide case.

2) becomes stable for the growth vs proton concentration of species a being negative and for b being positive at p=0. Moreover, has to be negative. Since is per definition positive and can only become negative if c_b_ becomes negative. 2) can only become stable for species b lowering the proton concentration but at the same time showing positive growth at low (p=0) proton concentration, whereas species a dies at p=0.

3) becomes stable for the growth vs proton concentration of species a being negative and for b being positive at p=. has to be positive, which can only be achieved, with c_b_ being positive and thus species b has to increase the proton concentrations but at the same time tolerate high proton concentrations, whereas a has to die at high proton concentrations.

4) becomes stable for the growth vs proton concentration of species a being positive and for b being negative at p=. has to be negative which means c_a_ has to be negative.

5) becomes stable for the growth vs proton concentration of species a being positive and for b being negative for p=0. has to be positive which means c_a_ has to be positive.

A combination of 2) and 4) or 3) and 5) results in the bistability case, where two fixed points exist but the stability of the fixed points is decided by the pH.

6) becomes stable for the growth vs proton concentration of both species being positive at p=0. That means both species have to survive low proton concentrations. Moreover, the system has to fulfill the condition c_b_<-c_a_/d .

7) becomes stable for the growth vs proton concentration of both species being positive at p=. That means both species have to survive high proton concentrations. Moreover, the system has to fulfill the condition c_b_ >-c_a_/d .

6) and 7) correspond to the 'trivial' case where both species have a similar growth vs proton concentration behavior .

The Eigenvalues for the remaining cases are more complicated:

8)

9)

10)

11)

However, they all have in common that the first eigenvalue corresponds to the negative values of the growth vs proton concentration curve at the fixed point. These values get only negative when the growth of species a or b respectively is positive at the fixed point. The second eigenvalue always is the negative of the third. Thus if one of the eigenvalues is negative the other one becomes positive and thus the fixed point is unstable. The fixed points thus only can be stable when real part of the second and third eigenvalues become zero. Since the first factor of these eigenvalues is mostly non zero, a real part of zero is most easily reached by setting the expression under the square-root equal or smaller zero. This can on the one hand just be achieved for a certain set of parameters and one th other hand causes in many cases imaginary parts of the eigenvalues to be nonzero, which show the possibility of oscillations. Both properties can indeed be found in the the simulation (Fig. 3d).

**Stability analysis for a generalized growth vs proton concentration function**

To test in how far the choice of the growth vs proton concentration curve influences the properties of the system we replaced this last factor in equation (3) we wrote a separate differential equation for species a and b and used general functions f(p) and g(p) for the growth vs proton concentration curves.

$\frac{\partial n_{a}}{\partial t}=n_{a}\left( 1-n_{a} \right)f\left( p \right)$ **(5)**

$\frac{\partial n_{b}}{\partial t}=n_{b}\left( 1-n_{b} \right)g\left( p \right)$ **(6)**

$\frac{\partial p}{\partial t}=\left( \alpha n_{a}+n_{b} \right)\left( \beta p\left( \gamma-p \right) \right)$  **(7)**

The corresponding fixed points are:

1) n_a_=0, n_b_=0

2) n_a_=0, n_b_=1, p=0

3) n_a_=0, n_b_=1, p=

4) n_a_=1, n_b_=0, p=0

5) n_a_=1, n_b_=0, p=

6) n_a_=1, n_b_=1, p=0

7) n_a_=1, n_b_=1, p=

8) n_a_=-1/, n_b_=1, f(p)=0

9) n_a_=1, n_b_=-, g(p)=0

The corresponding eigenvalues at the fixed points are:

1)

2)

3)

4)

5)

6)

7)

8)

9)

Again for the fixed points 1)-7) the stability at the fixed points are determined by the parameters , and and the values of the functions f(p) and g(p) at the fixed points, which are not depending on the exact shape of those functions. The fixed points 8) and 9) become stable when the real part of the first two eigenvalues becomes zero which is obtained by having non positive values under the squareroots, which is true only for rather specific sets of parameters and usually causes imaginary parts and thus oscillations of the system.

**Fuzzy logic model to predict interaction outcomes**

Using differential equations to describe the growth and pH change of/by the bacteria implies a certain mechanistic understanding that we do not necessarily have ( therefore the model above should just be seen a qualitative description. To overcome this issue we present a fuzzy logic based model here. Fuzzy logic allows to do math based on qualitative rules and thus produces rough estimated based on rough inputs in combination with semi-quantitative rules.

The approach describes the microbes and environment as a network with two or three nodes for the one and two species systems respectively. The nodes represent the cell densities of the one or two species and the pH of the environment respectively. Species nodes are connected to the environment and *vice versa* as shown in Fig. 3A. This way the pH node gets the cell density of one/both species as input and results in a pH change, whereas the species nodes get the pH as input and result in a cell density change. This process is repeated iteratively until a stable result is obtained. The calculation of the species density and pH change are done by fuzzy logic. The input is fuzzified with the membership functions shown in Supplementary Fig. 14. The change in pH and cell density are calculated with the rules shown in Tables 1 and 2 and added to the old pH and cell density values. If the pH or cell density are outside the minimal/maximal values (pH<0 or pH>14, cell density<0 or cell density >8) the pH and cell densities are set to the maximum or minimum. The IF-THEN rules were applied with Mamdani inference and the defuzzification to scalar values was done with centroid method [1].

The membership functions for the input (pH and cell densities), the parameters (preferred pH and direction of pH change) and the output (overall change in pH and cell densities) are shown in Supplementary Fig. 14.

The set of rules is shown in Supplementary Table 1 and 2.

With this rules the processing of the system was calculated according to the following pseudo-code. The relative strength allows to tune how much stronger one species can change the pH compared to the other. This effect matters for the case of murder suicide (see Supplementary Figure 15C).

The results of the fuzzy based simulations are shown in Supplementary Fig. 15. The final presence or absence of a species is shown for different initial cell densities or cell density ratios for the co-culture and different initial pH values.

The code for the simulations is provided as S4_Data.

**References:**

1. Ross, T. J. *Fuzzy Logic with Engineering Applications*. (John Wiley & Sons, 2009).
